# Evolutionary and immune microenvironment dynamics during neoadjuvant treatment of oesophagael adenocarcinoma

**DOI:** 10.21203/rs.3.rs-2738048/v1

**Published:** 2023-04-13

**Authors:** Melissa Barroux, Jacob Househam, Eszter Lakatos, Tahel Ronel, Ann-Marie Baker, Henrike Salié, Max Mossner, Kane Smith, Chris Kimberley, Salpie Nowinski, Alison Berner, Vinaya Gunasri, Mamix Jansen, Giulio Caravagna, Katja Steiger, Julia Slotta-Huspenina, Wilko Weichert, Markus Alberstmeier, Benny Chain, Helmut Friess, Bertram Bengsch, Roland Schmid, Jens Siveke, Michael Quante, Trevor Graham

**Affiliations:** Medical Clinic and Polyclinic II, Klinikum rechts der Isar, Technical University of Munich; Institute of Cancer Research; Institute of Cancer Research; Barts Cancer Institute, Queen Mary University of London; Institute of Cancer Research; University Medical Center Freiburg; Institute of Cancer Research; Institute of Cancer Research; Barts Cancer Institute, Queen Mary University of London; Institute of Cancer Research; Barts Cancer Institute, Queen Mary University of London; Institute of Cancer Research; UCL Cancer Institute: University College London Cancer Institute; University of Trieste; Institute of Pathology, School of Medicine, Technical University of Munich; Institute for Pathology, Technische Universität München; Technische Universität München; University Hospital, Ludwig-Maximilians-Universität (LMU) Munich; UCL; Department of Surgery, Klinikum rechts der Isar, Technical University of Munich; University Medical Center Freiburg; Medizinische Klinik und Poliklinik II, Klinikum rechts der Isar, Technische Universität München; German Cancer Consortium (DKTK) partner site Essen and Institute for Developmental Cancer Therapeutics (BIT), University Hospital Essen at the University Duisburg-Essen, Germany; University of Freiburg; Institute of Cancer Research

## Abstract

Locally advanced oesophageal adenocarcinoma (EAC) remains difficult to treat because of common resistance to neoadjuvant therapy and high recurrence rates. The ecological and evolutionary dynamics responsible for treatment failure are incompletely understood. Here, we performed a comprehensive multi-omic analysis of samples collected from EAC patients in the MEMORI clinical trial, revealing major changes in gene expression profiles and immune microenvironment composition that did not appear to be driven by changes in clonal composition. Multi-region multi-timepoint whole exome (300x depth) and paired transcriptome sequencing was performed on 27 patients pre-, during and after neoadjuvant treatment. EAC showed major transcriptomic changes during treatment with upregulation of immune and stromal pathways and oncogenic pathways such as KRAS, Hedgehog and WNT. However, genetic data revealed that clonal sweeps were rare, suggesting that gene expression changes were not clonally driven. Additional longitudinal image mass cytometry was performed in a subset of 15 patients and T-cell receptor sequencing in 10 patients, revealing remodelling of the T-cell compartment during treatment and other shifts in microenvironment composition. The presence of immune escape mechanisms and a lack of clonal T-cell expansions were linked to poor clinical treatment response. This study identifies profound transcriptional changes during treatment with limited evidence that clonal replacement is the cause, suggesting phenotypic plasticity and immune dynamics as mechanisms for therapy resistance with pharmacological relevance.

## Introduction

Oesophageal cancer is the sixth most common cause of cancer-related death worldwide, with a median overall survival of <1 year^1^. Incidence rates for oeshophageal adenocarcinoma (EAC) have risen sharply and it is now the predominant subtype in high-income countries^2^. Patients with locally advanced EAC are treated with neoadjuvant chemotherapy (CTx) or radiochemotherapy (RCTx) followed by surgical resection^3^. Although neoadjuvant treatment confers a survival benefit over resection alone, 50-60% of tumours are resistant to neoadjuvant therapy, leading to an overall poor outcome with a 5-year survival of 12.6%^3^. There is currently no way to determine who will benefit from neoadjuvant treatment.

Genetic sequencing studies have examined the genetic alterations in EACs during treatment in order to understand evolutionary dynamics that may lead to treatment resistance^4–6^. The translational goal of these studies is to translate measures of the evolutionary dynamics into treatment-predictive biomarkers. However, there is growing evidence that genetic evolution alone does not fully explain resistance evolution^7^. Tumour plasticity, defined as cellular phenotype changes in the absence of underlying genetic change, is associated with treatment resistance in multiple cancer types^8–10^. Furthermore, there is mounting evidence that the efficacy of chemotherapies also relies on activating antitumor immune responses^11^. Measures of the tumour microenvironment (TME) are an important predictor for the response to neoadjuvant treatment and for future perspectives in regard to clinical trials e.g. KEYNOTE-585, which evaluates combined checkpoint and chemotherapy in the perioperative setting in EAC patients^12^.

There is a major practical challenge in collecting longitudinal samples over space and time from solid tumours. Here we have performed multi-region, multi-timepoint whole exome sequencing (WES) at high depth (mean 300x) of locally advanced EAC to identify mutations and track clonal dynamics across time and space, with matched RNA-sequencing to characterize phenotype changes, and imaging mass cytometry (IMC) and T-cell receptor (TCR) sequencing to characterize immune infiltrates and stromal cell dynamics. Our comprehensive analysis provides a holistic multi-omic view of the dynamic changes in the tumour and its microenvironment through neoadjuvant treatment.

## Results

### Longitudinal multi-omic profiling in 10 Non-Responder EAC and 17 Responder EAC

EAC specimens were obtained from the prospective clinical DKTK-funded MEMORI trial^13^. Patients underwent baseline (18)F-FDG PET followed by 1 cycle of neoadjuvant platin-based chemotherapy, and clinical response was assessed at day 14-21 by (18)F-FDG PET. Responders (REs) to the initial treatment continued to receive chemotherapy, whereas non-responders (NRs) were switched to intensified RCTx (for detailed information see methods part 1).

**Figure 1:**
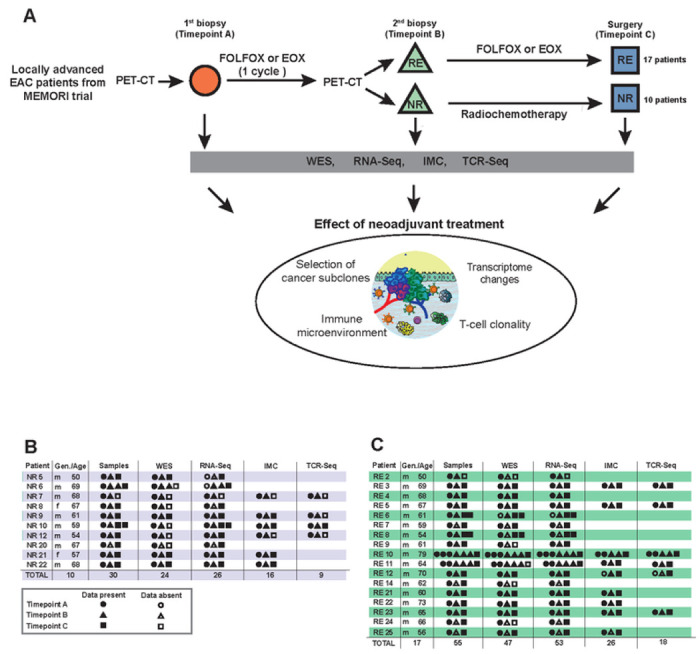
Experimental workflow and overview of the study cohort. **(A)** Flowchart summarising patient treatment and study design, including respective neoadjuvant treatment, sample acquisition and analyses. FOLFOX: Oxaliplatin and 5-FU, EOX: epirubicin, oxaliplatin, capecitabine, RE: Responder, NR: Non-Responder, WES: whole exome sequencing, RNA-Seq: RNA-Sequencing, IMC: imaging mass cytometry, TCR-Seq: T-cell receptor sequencing. Overview of the study cohort of Non-Responders **(B)** and Responders **(C)** with indication of samples present for each type of data analysis. Samples from different timepoints are indicated with different shapes. RE: Responder; NR: Non-Responder; Gen: gender, Age: age at diagnosis; WES: whole-exome sequencing; RNA-Seq: RNA-Sequencing, IMC: imaging mass cytometry, TCR-Seq: T-cell receptor sequencing.

Longitudinal molecular analysis was performed on 10 NRs and 17 REs (Fig 1A). From most patients three cancer samples were collected: one pre-treatment biopsy (Timepoint A), one biopsy collected after the first cycle of platin-based chemotherapy (Timepoint B) and the surgical cancer resection specimen sample after completion of neoadjuvant treatment (Timepoint C) (Fig 1A, B and C). For two patients (RE10 and RE11) we performed multi-timepoint and multi-region sampling (≥ 6 samples per patient).

After clinical and histopathological review (supplemental table 1) all samples were micro-dissected (see methods section 2.1), DNA extracted and subjected to whole exome sequencing (24 tumour samples from NRs and 47 tumour samples from REs; at mean coverage of 300x (range 44-478x) with matched germline control DNA from blood sequenced at mean coverage of 282x (supplemental table 2)). SNV, indel and copy number alteration (CNA) calls were derived from sequencing data, along with estimations of tumour cellularity. Tumour cellularity showed no significant difference in our WES data between timepoints (supplemental fig 1A). Matched bulk 3’RNA-Seq was performed on 26 samples from NRs and 53 samples from REs (supplemental table 3). Dynamics in immune infiltrates during treatment were analysed with IMC using a comprehensive panel of 14 immune, 3 epithelial, 1 stromal, 1 proliferation and 2 nuclear marker (supplemental table 4). IMC was completed on 24 regions of interests (ROIs) from 16 NR samples and 42 ROIs from 27 REs samples (Fig 1B and C). Bulk TCR-sequencing was performed on selected EACs with high RNA quality and satisfactory immune cell infiltration according to our CIBERSORT and Consensus^TME^ immune deconvolution. 18 samples from REs and 9 samples from NRs were eligible for TCR-Seq (Fig 1B and C).

### EAC shows limited evidence for strong clonal selection during neoadjuvant treatment

We assessed genetic changes (SNVs and CNAs) in responders and non-responders (Fig 1A, B and C). We observed an average mutational burden of 23.09 SNVs/Mb (range 7.5 - 127.7 SNVs/Mb) in treatment naive EAC, which is consistent with previously described mutation burden in EAC, accounting for our higher depth of coverage^14,15^.

**Figure 2:**
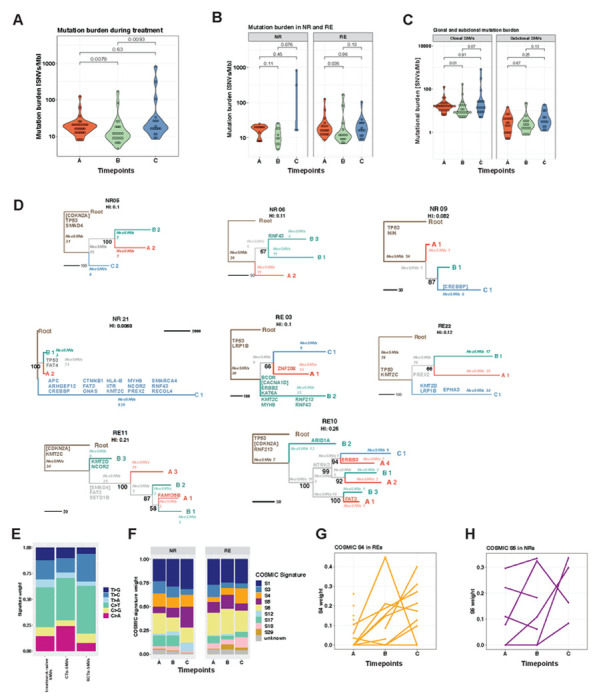
Evolutionary dynamics of mutations during neoadjuvant treatment in EAC. **(A-C)** Violin plots showing the distribution of mutational burden during neoadjuvant treatmentfor all patients (A), stratified by treatment response (B) and stratified by clonality of mutation (C). Mutations of each sample were classified as clonal or subclonal based on the copy number and cellularity adjusted cancer cell fraction. P values in panels A-C are calculated by the Wilcoxon test. **(D)** Selected phylogenetic trees with clade length indicating the number of shared mutations between samples. Timepoint of samples are annotated at the tip of the clades with the letters A-C. Numbers at the nodes indicate bootstrap values. EAC drivers harbouring SNVs (without brackets) or indels (in squared brackets) and number of neoantigenic SNVs are annotated on the clades of the trees. RE: Responder, NR: Non-Responder, HI: homoplasy index, NeoSNVs: Neoantigenic single-nucleotide variant. **(E)** Proportion of SNV types in treatment-naive SNVs (left), SNVs occurring under chemotherapy (middle) and SNVs occurring under RCTx (right). SNV: single nucleotide variant, CTx: platin-based chemotherapy, RCTx: radiochemothearapy. A: adenine, T: thymine, C: cytosine, G: guanine. **(F)** Proportion of COSMIC Signatures in Non-Responders and Responders during treatment (COSMIC signature calling was limited to those with a weight > 5% in the respective groups). **(G)** Line graph showing changes in the proportion of COSMIC Signature 4 in Responders during treatment **(H)** Line graph showing changes in the proportion of COSMIC Signature 5 in Non-Responders during treatment. For multi-region samples means were plotted for each timepoint.

The overall number of SNVs between pre-and post-treatment samples showed no significant difference (Fig 2A), but we noted that two NRs showed large increases in SNV burden after radiation (Fig 2B) suggesting radiation-induced mutagenesis. Further, we assessed changes in mutation clonality. If treatment drove clonal selection, we might expect to see increased clonal and/or subclonal mutational burden between pre- and post-treatment. However, no significant difference was detected for either mutation class (Fig 2C).

To probe clonal dynamics, we constructed phylogenetic trees using SNVs for 15 patients with multi-timepoint and multi-region samples (≥ 3 samples) (Fig 2D, supplemental fig 1B)^16^. The majority of phylogenetic trees were ‘balanced’ with similar clade lengths for all samples. We observed progressive clonal sweeps during neoadjuvant treatment in only one sample (NR9), wherein the clade from Timepoint C arose from B, and B arose from Timepoint A (Fig2D, supplemental fig 1B). In all other cases, samples from timepoint C were either most similar to precursor clones of samples from timepoints A and B (e.g. NR21 and RE22), or most similar to a clone detected at timepoint A (e.g. RE10). A parsimonious explanation of these observations is that the cancer was a mosaic of clones, and inferred clonal relationships between timepoints and/or spatial samples were determined by spatial sampling rather than by a clonal sweep driven by treatment.

**Figure 3:**
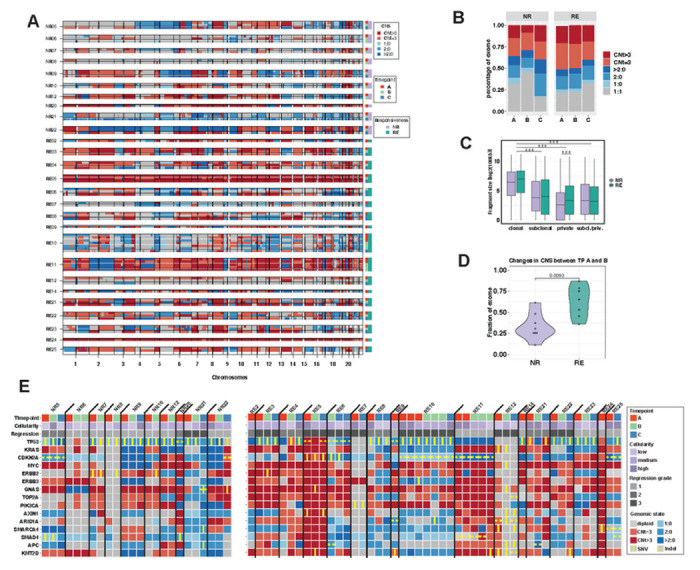
Dynamics in copy number alterations (CNAs) during neoadjuvant treatment. (A) Plot showing genome-wide copy number state. Each row represents a sample, with samples from the same patient grouped together and patient ID is annotated on the left. Treatment response and timepoint of each sample are annotated on the right. Dashed vertical lines indicate the centromere of each chromosome and continuous vertical lines are separating different chromosomes. NR: Non-Responder, RE: Responder, CNS: copy number state, CNt: copy number **(B)**Percentage of altered exome in RE and NR during neoadjuvant treatment. **(C)** Fragment size of clonal, subclonal and private copy number alterations. In patients with only 2 samples available, no distinction between subclonal and private could be made and therefore CNAs were summarized in “subclonal/private” category. *** indicates p < 0.001 by the Wilcoxon test **(D)**Fraction of exome with changing copy number state between Timepoint A and B in NR and R. P values are calculated by the Wilcoxon-test. CNS: copy number state, TP: Timepoint, RE: Responder, NR: Non-Responder **(E)** Plot shows genetic alterations, including copy number alterations, SNVs and indels for putative cancer driver genes identified by IntOGen©. Each vertical column represents a sample. Samples of the same patients are grouped together and patient ID is annotated at the top. Information on timepoint, cancer cellularity and the patient’s pathological regression grade treatment are found in the top three rows. The following rows show information on genetic alterations in EAC driver genes. Cellularity was defined as low (15-30%), medium (31-60%), or high (61-100%). Regression grades were evaluated by a pathologist according to Becker regression classification. SNV: single nucleotide variant.

Patients NR21 (Fig 2D) and NR22 (supplemental fig 1B) who accrued many mutations during RCTx were outliers with long branches for timepoint C (resection post-treatment), potentially indicating the outgrowth of a highly mutated subclone. Indeed, we observed a highly fragmented genome in our copy number analysis post RCTx (Fig 3A) and a dN/dS analyses to detect evidence of clonal selection indicated RCTx-induced random DNA damage (dN/dS<1) rather than SNVs undergoing positive selection (supplemental table 5).

EAC driver mutations as listed by IntOGen^®17,18^ and neoantigenic SNVs were annotated on each phylogenetic tree (Fig 2D and supplemental fig 1B). Known high-frequency driver events such as *TP53* and *CDKN2A* were mostly truncal on the phylogenetic trees, indicating that these drivers persisted through treatment. *TP53* was mutated by SNVs in 17 patients (Fig 2D, 3E, supplemental fig 1B) and indels in 4 out of 27 patients (Fig 2D, 3E, supplemental fig 1B) from which 13 out of 22 patients with ≥ 2 samples showed clonal *TP53* alterations present in all samples. *CDKN2A* showed SNVs in 2 patients (Fig 3E, supplemental fig 1B) and harboured indels in 5 patients (Fig 2D, 3E) with clonal genetic *CDKN2A* alterations in 5 out of 22 patients with ≥ 2 samples (Fig 2D, 3E), consistent with prior reports on EAC genomes^14,15,19,20^. In our phylogenetic trees (Fig2D, supplemental fig 1B), additional non-silent mutations in known driver genes events were detected at the end of neoadjuvant treatment in 7 out of 12 patients with sequenced samples from pre- and posttreatment timepoints (timepoint A and C present). These new putative driver mutations were high frequency driver (genes that are detected as drivers in >5% of samples in Intogen cohorts) in only 1 patient (CDKN2A in NR22). Therefore, 11/12 patients with sequenced samples from pre- and posttreatment timepoints had either no new putative drivers post-treatment, or low-frequency drivers, suggesting no major changes in the clonal make-up for the large majority of patients during neoadjuvant treatment.

Distinct treatment regimens have been associated with specific mutational footprints^21^. We assessed the specific mutagenic consequences of therapy on our cohort. SNVs from our phylogenetic trees were classified into treatment naïve SNVs, SNVs acquired during chemotherapy exposure (CTx-SNVs) and SNVs acquired during RCTx exposure (RCTx-SNVs). In CTx-SNVs a significant increase in C>A mutations was observed compared to treatment-naive SNVs (Wilcoxon rank-sum test *P*-value=0.03) (Fig 2E), which is consistent with previously described platin-induced mutation signatures^4,5,21^. RCTx-SNVs showed a significant increase in T>C (Wilcoxon rank-sum test *P*-value=0.009) (Fig 2E). Across subgroups from different treatment response and timepoints, we detected nine prevalent COSMIC signatures (Fig 2F), of which eight have been previously identified in oesophageal cancer^6,22–24^. Signature 12, which had not been previously described in EAC, was at very low activity in treatment naïve samples, but increased during RCTx and might therefore represent a treatment induced rather than EAC specific signature. REs showed an non-significant increase in S4 signature during treatment (Wilcoxon rank-sum test p-value=0.036 and p-adj=0.34 between Timepoints A and C), which is dominated by C>A changes and thus most likely oxaliplatin driven^21^ (Fig 2F+G). NRs depicted a non-significant increase of radiotherapy associated S5 signatures^25^ after exposure to RCTx (Wilcoxon rank-sum test p-value=0.14 and p-adj=0.6 between Timepoints A and C) (Fig 2F+H).

### Copy number evolution through treatment

EACs genomes are dominated by a high burden of CNAs in the majority of cases^15,22,26^. Our WES data from 17 REs and 10 NRs allowed us to comprehensively track CNAs in coding regions throughout neoadjuvant treatment. We observed our cases had a high CNA burden with a median of altered genome of 71.4% (range 1.1 - 100%) (Fig 3A). Copy number (CN) amplifications were seen frequently on chromosomes 1q, 3q, 5p, 7p, 8q, 19, 20, while losses or loss of heterozygosity (LOH) predominated on chromosomes 3p, 4q,5q, 9p and 17p, consistent with previous work^4,18,22^(Fig. 3A).

Treatment-naive tumours from REs and NRs displayed similar proportions of amplifications, deletions, loss and LOH regions (Pearson’s Chi-squared test >0.05) (Fig 3B) to post-treatment samples. Chemotherapy did not increase the overall percentage of altered genome, whereas RCTx, known to cause broad single or double strand breaks, led to a slightly higher proportion of altered genome in samples post-RCTx compared to pre-treatment samples, though the difference was not significant (t-test p-value=0.09) (Fig 3B). The radiation-associated changes were dominated by exome-wide small fragment losses or “scrambled” chromosomes (Fig 3A) in a manner previously described in radiation-induced CNA and small fragment deletions^25,27^. We analysed the size of clonal, subclonal and private CN fragments in RE and NR and observed that genome fragments that change their CN state during treatment were significantly smaller than clonal fragments (Fig 3C). Furthermore, we observed significantly smaller fragment sizes of private CNAs in NRs than in REs (t-test *p*-value < 0.001), which is consistent with the radiation-induced DNA damage in NRs.

We examined if dynamics in CNAs correlated with early treatment response in EAC. REs showed significant changes in copy number alteration burden with a mean of 63% (range 36-87%) of exome changing its CNA status (to any CN state) after the first cycle of chemotherapy, whereas copy number states in NRs were more stable with a mean of 33% (range 11-61%) of exome altering CNA state (Wilcoxon rank-sum test p-value 0.009; Fig 3D). We hypothesize that NRs have genomes that confer pre-existing resistance to the effect of neoadjuvant chemotherapy, whereas REs have patterns of CNAs that make the cells more sensitive to chemotherapy, and so the first dose of chemotherapy prompts further CNA evolution.

### Genetic and transcriptomic dynamics In EAC drivers reflect treatment response

We focused on genetic alterations in the 108 genes suggested to be drivers of EAC evolution (supplemental table 6)^17,18^. Genetic alterations in 15 common EAC driver genes across are shown in Fig 3E (genetic alterations in all 108 IntOgen drivers are shown in supplemental fig 2 and 3). In 19 out of 27 patients, we observed that *TP53* LOH or loss was associated with SNVs or indels of the same gene, presumably leading to a loss of normal function of *TP53*. *CDKN2A* showed CN loss or LOH in 20 out of 27 patients, which cooccurred with SNVs or indels in 5 patients, consistent with the nature of the disease^14^. We further observed amplifications containing *MYC*, *KRAS*, *ERBB2*, *ERBB3*, *GNAS*, *TOP2A* and *PI3KCA* and deletion of *SMAD4*. Interestingly, CNAs in driver genes were mostly present before treatment and persisted in their CNA category (amplification, LOH, loss) during treatment, although the exact CNS could vary (Fig 3E). Both RE and NRs showed no significant change in the burden of genomic alterations of driver genes during treatment (supplemental fig 4A-F). REs showed a mean of 89.3 genetically altered putative driver genes at Timepoint A, 92.4 at B and 79.4 at Timepoint C (supplemental fig 4B). NRs had a mean of 77.1 genetically altered putative drivers at Timepoint A, 65.4 at B and 91.0 at Timepoint C. Moreover, we did not detect any specific cancer gene that was enriched for CNAs, SNVs or indels in the RE versus NR group (Fig 3E, suppl. fig 2 and 3). We note that work in colorectal cancer has shown that mutations that are drivers are “on average” may not be a driver in a particular cancer type^10^ and so we do not claim that all of these mutations are necessarily driving EAC evolution.

We next explored gene expression changes in high frequency drivers using our matched RNA-seq data. Significant expression changes were observed for most driver genes: expression tended to increase for *GNAS*, *PIK3CA*, *SMAD4*, decrease for *ARID1A*, *CDKN2A*, *ERBB2*, *ERBB3*, *TOP2A*, *SMARCA4*, *KRAS*, *KMT2D* and was stable for *TP53*, *MYC*, *APC* and *AXIN1* (supplemental fig 4G). We tested whether these expression changes were likely driven by underlying genetic copy number alteration, finding little evidence that copy numbers drove expression. Copy number state and RNA expression were strongly positively correlated for GNAS and moderately correlated for *SMARCA4* and *TP53* (supplemental fig 7), and poor correlations were observed for ARID1A, APC, *AXIN*, *CDKN2A*, *ERBB2*, *KMT2D*, *SMAD4*, *TOP2A*, and no correlations were observed for *ERBB3*, *KRAS*, *MYC* *and PIK3CA* (supplemental fig 5A). Correlation analyses between SNVs in driver genes and their expression, was only possible for TP53 and CDKN2A, as analyses for other drivers were underpowered. We observed significantly higher CDKN2A expression in CDKN2A mutated samples and significantly lower p53 expression in TP53 mutated samples (supplemental fig 5B).

In summary, we observed major changes in gene expression of purported driver genes. However, these changes were rarely explainable by underlying genetic alterations or change.

### Neoadjuvant treatment alters the EAC transcriptome

**Figure 4:**
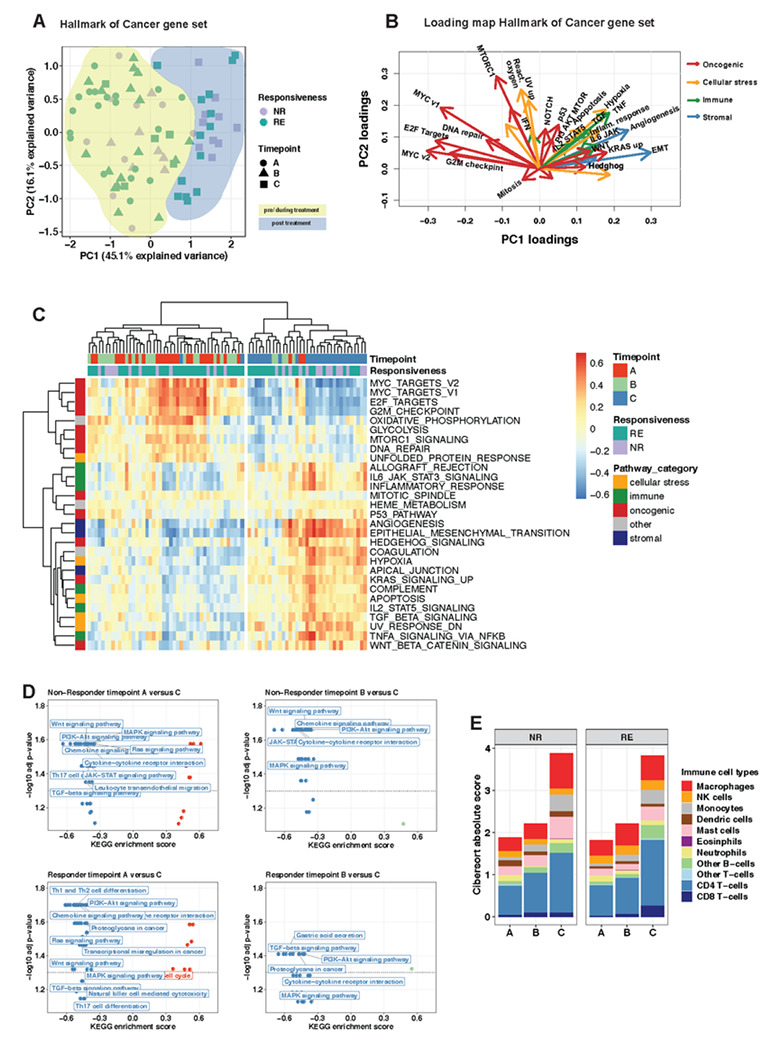
Neoadjuvant treatment leads to profound changes in gene and pathway expression in EAC. **(A)** Principle component analysis of single sample gene set enrichment analysis of cancer hallmark gene sets PC: principal component. **(B)** Principal component feature loadings (magnitude and direction) from PCA in A. Vectors are colored according to their biological classification of cancer hallmark gene sets. **(C)** Hierarchical clustering with heatmap showing the significantly differentially expressed pathways between the two clusters (left cluster is predominantly samples from timepoint A/B and right cluster is predominantly timepoint C). **(D)** Enrichment in KEGG pathways in Non-Responders between Timepoint A and C (upper left), and between Timepoint B and C (upper right), and in Responders between Timepoint A and C (lower left), and between Timepoint B and C (lower right). Dotted line indicates significance level of p_adj_ < 0.05. **(E)** Plot shows immune cell composition based on CIBERSORT analysis in Responders and Non-Responders during neoadjuvant treatment.

We assessed gene and pathway expression changes during neoadjuvant treatment using our matched RNA-seq data from 26 samples from 10 NRs and 53 samples from 17 REs. Principle component analysis (PCA) using hallmark pathways^28^ separated samples from Timepoint A and B from samples at the end of the neoadjuvant treatment (Fig 4A), whereas samples from REs and NRs were intermixed. Hallmark pathways were grouped into “classes” according to their biological function (“oncogenic”, “immune”, “stromal”, “cellular stress”)^29^. PCA loading assessment showed that samples from Timepoint A and B were enriched for oncogenic pathways, whereas samples from Timepoint C were enriched in immune and stromal pathways and selected oncogenic pathways such as *KRAS*, *Hedgehog* and *WNT* signalling (Fig 4B). We identified genes that were on average significantly differentially expressed hallmark pathways between pre/early-treatment samples and samples at the end of neoadjuvant treatment, and performed hierarchical clustering of all samples using these genes. Expectedly, and consistent with PCA analysis, the dendrogram separated into two groups (Fig 4C): Group 1, containing most samples from Timepoint A/B, was enriched for “oncogenic” acting pathways, such as *MYC,* cell cycle associated pathways, DNA repair *and MTORC* signalling (Fig 4C), Group 2, including mostly samples from Timepoint C, showed significant enrichment of EMT and stemness associated *WNT* signalling and promoters of stem cell like state such as *TGF-β* signalling and hypoxia. Moreover, immune pathways such *IL6-,* *IL2*-signaling and inflammatory response and oncogenic *KRAS-, Hedgehog-* and *p53*-signaling were significantly enriched in the post-treatment group (Fig 4C).

When examining pathway enrichment in early and late response in the different treatment regimens, neither REs nor NRs showed significant changes in pathways between Timepoint A and B .(supplemental table 7 for REs, no KEGG enrichments found for NRs). However, REs showed some evidence of early upregulation in immune activation pathways (q-value=0.24) (supplemental table 7), whereas NRs did not. Of note, samples from REs and NRs showed enrichment of same pathways after completion of neoadjuvant treatment with upregulation of *WNT-*, *PI3K*, *RAS*-, *MAPK*-, *JAK-STAT* and *Hedgehog* signalling and immune related pathways (Fig 4D), indicating that both chemotherapy and RCTx lead to fundamental but similar transcriptomic changes in EAC, potentially as acute wounding/wound healing response.

We used immune deconvolution tools to delineate cellular composition changes from RNA-Seq data^30,31,32^. Using Cibersort^30^ deconvolution, we observed a gradual increase in absolute numbers of immune cells during neoadjuvant treatment in REs and NRs (Fig 4E). Specifically, we observed a doubling of immune cell infiltrates in samples after completion of neoadjuvant treatment compared to pre-treatment samples, which might be due to lower tumour cellularity in bulk RNA sequencing data from post-treatment samples. Proportions of individual immune cell types stayed stable during treatment (Fig 4E, supplemental fig 6A), in which CD4 and CD8 cells represented the most abundant immune cell fraction (40-50% of all immune cells). Other deconvolution tools (Consensus^TME^, Syllogist)^31,32^ confirmed the gradual increase in immune infiltrates during treatment (supplemental fig 6B, C) but there was variability amongst tools in the estimated proportions of immune cell types (supplemental fig 6B).

In summary, gene expression programs, relevant for cancer biology and the surrounding immune microenvironment, significantly changed during treatment. Marked transcriptomic changes with enrichment of developmental programs such as EMT, stemness-associated *WNT* signalling and promoters of stem cell like state such as *TGF-β* signalling and hypoxia were observed, despite infrequent clonal replacement. Our data therefore suggest that cancer cells alter their phenotype without clonal replacement and that phenotypic plasticity might underly those phenotype alterations.

### Immune escape correlates with treatment response

Neoantigenic mutations can induce antitumor immune response, through the presentation of altered peptides that are being recognized as ‘foreign’ by the immune system^33,34^. With the recent introduction of combined immuno-chemotherapy for treatment of advanced EAC^35^, we focussed on understanding evolutionary dynamics of neoantigentic mutations, immune escape mechanisms and the response of the surrounding immune microenvironment.

**Figure 5:**
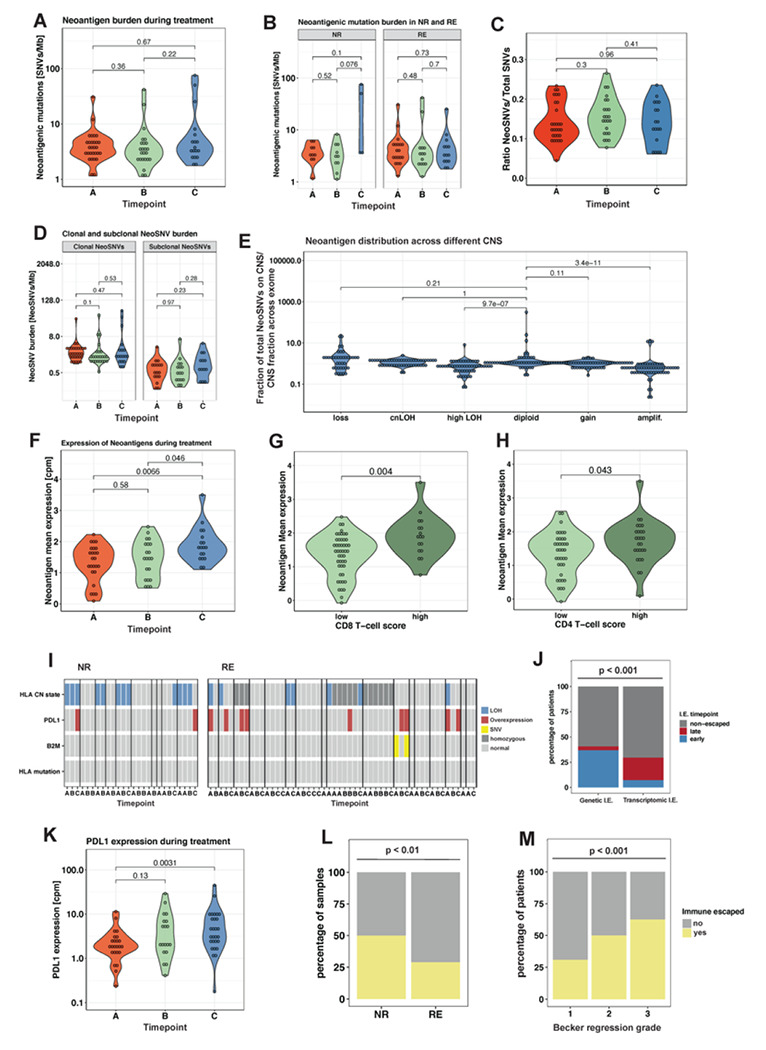
Increasing immune escape during neoadjuvant treatment correlates with poor treatment response. **(A-D)** Violins showing the neoantigenic mutational burden during neoadjuvant treatment in all samples (A), stratified by treatment response (B), expressed as a proportion of total SNVs (C), and stratified by clonality (D). **(**Clonal and subclonal neoantigenic mutational burden during neoadjuvant treatment. Mutations of each sample were classified as clonal or subclonal based on the copy number and cellularity adjusted CCF. CNS: copy number state **(E)** Distribution of neoantigenic SNVs based on their copy number-states. The copy-number normalised proportion of neoantigenic SNVs in each CN segment was calculated. **(F)**Expression of neoantigens during treatment. **(G-H)** Neoantigen expression in EAC according to immune infiltration score for **(G)** CD8 T-cells and **(H)** CD4 T-cells. **(I)** Presence of HLA LOH, PD-L1 overexpression and B2M mutations in individual samples from Non-Responders (left) and Responders (right). Samples from individual patients are separated by vertical black lines. PD-L1 overexpression was defined as PD-L1 expression >2 standard deviations from the mean of all treatment naive samples. **(J)** Proportion of immune escaped patients. Genetic immune escape refers to mutations or LOH in HLA or B2M mutations, whereas PD-L1 overexpression represents transcriptomic immune escape. P value calculated by the chi-square test. **(K)** PD-L1 expression during neoadjuvant treatment. **(L-M)** Proportion of immune escaped patients stratified by treatment response (L) and pathological regression grades (M). P value calculated by the chi-square test. P values in all panels are calculated by the Wilcoxon test, unless stated otherwise.

Neoantigens were called from WES data on 24 samples from 10 NRs and 47 samples from 17 REs. We did not observe any significant changes in the neoantigenic mutation burden during neoadjuvant treatment (Fig 5A). However, NRs showed a slight increase in neoantigenic mutation burden after being switched to RCTx (Fig 5B) consistent with the observed increase in total mutational burden (Fig 2B).

To understand the selection mechanisms of neoantigenic mutations, we examined if treatment enhances the immune response against the tumour cells. Depletion of neoantigenic mutation burden relative to the overall mutation burden is a well-established signature of immune-editing^36,37^. Our data showed a stable ratio between immunogenic and total mutations during neoadjuvant treatment (Fig 5C), indicating no enhanced negative selection of neoantigenic mutations during treatment. We further did not observe a significant difference in the subclonal neoantigenic mutational burden between pre- and posttreatment samples (Fig 5D) and for most patients comparable amounts of neoantigenic SNVs on the clades of their phylogenetic tree (Fig 2D), which did not indicate removal of neoantigenic subclones during treatment.

Next, we explored the relationship between CNAs and neoantigens. We hypothesised that gains of alleles carrying antigenic mutations were likely to experience negative selection, and so expected that gained alleles would be relatively depleted for neoantigens compared to not-gained alleles. To test this, we calculated the copy-number normalised proportion of neoantigens in each CNA segment. Diploid regions showed a ratio of ~ 1 (Fig 5E). Gains (balanced and unbalanced) were significantly depleted for neoantigens (Fig 5E) while regions of CN loss were weakly, but not statistically significantly, enriched for neoantigens (Fig 5E).

We hypothesized that rather than clonal evolution leading to removal of neoantigen clones, transcriptional downregulation of neoantigens during treatment might underlie immune evasion and potentially mediate treatment response. Counter to our hypothesis, we detected a significant increase in neoantigen expression during treatment (Fig 5F). High neoantigen expression correlated with high CD8 and CD4 cell infiltration (Fig 5G+H), suggesting that tumour cells with a high neoantigen expression were indeed recognized as ‘non-self’ and attracted immune cells. Consequently, we proposed that either EAC cells develop further immune escape mechanisms during treatment in order to avoid negative selection acting on their expressed neoantigens, or that the immune infiltrate becomes non-functional during treatment, and is not able to effectively eliminate tumour cells with high neoantigen expression. Both would be arguments for inclusion of immunotherapy in neoadjuvant treatment protocols.

We next examined the presence of immune escape mechanisms^38^ (LOH or mutations in HLA, B2M mutations, or the interaction with the immune microenvironment by programmed death-ligand 1 (PDL-1) overexpression) through treatment. Genetic immune escape mechanisms such as HLA LOH or B2M mutations were detected significantly more often prior to treatment start (early), whereas transcriptomic PDL1 overexpression occurred mostly during and after neoadjuvant treatment (late) (Fig 5I, J, K) possibly as a consequence of expression in stromal/immune cells. Consequently, genetic disruption of antigen presentation machinery did not appear to experience positive selection during neoadjuvant treatment, whereas expression of PDL-1, potentially as a mechanism of rapid adaptation, may be positively selected during treatment.

We examined the correlation between immune escape (either HLA LOH, B2M mutations or transcriptomic PDL1 overexpression present) and treatment response. Samples from NRs were significantly more often immune escaped than REs (Fig 5L). Further, patients with a poor pathological regression (Becker grade 3) were significantly more immune escaped (defined as a minimum of one immune-escaped sample) than patients with an intermediate (Becker grade 2) or good regression (Becker grade 1) (Fig 5M), suggestive of a role for the immune system in potentiating the chemotherapy treatment response. As immune escape correlated with a poor early response to chemotherapy and a poor overall response to neoadjuvant treatment, our data suggest it has an important role in treatment resistance, and a possibility that immune therapy could help to circumvent resistance to standard treatment.

### A distinct tumour microenvironment defines treatment response

**Figure 6:**
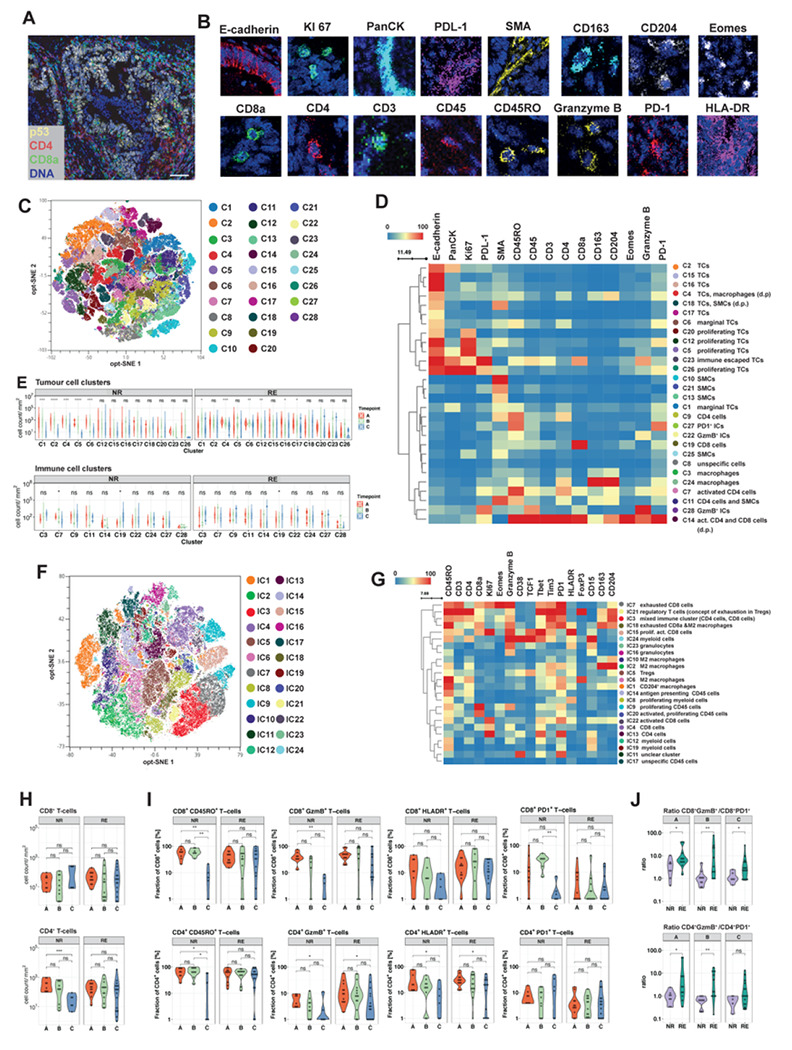
Highly multiplexed imaging mass cytometry analysis reveals different T cell phenotype dynamics in Non-Responders and Responders during treatment. **(A)** Representative image IMC staining of EAC tissue. Scale bar: 100μm. p53 (yellow), CD4 (red), CD8a (green) and DNA (blue) from IMC data channels are displayed. **(B)** Representative IMC images of each marker together with DNA (blue). **(C)** t-SNE visualization of the EAC tumour, stromal and immune map based on 28 identified cell clusters. **(D)** Heatmap visualization of marker expression in the 28 cell clusters. Normalized median marker expression is shown. TCs: tumour cells, SMCs: smooth muscle cells, ICs: immune cells, act: activated, d.p.: in direct proximity to each other, GzmB: Granzyme B **(E)** Cell cluster dynamics during treatment are shown for Responders and Non-Responders **(F)** t-SNE visualization of CD45^+^ cell map based on 24 identified cell clusters **(G)** Heatmap visualization of marker expression in the 24 CD45^+^ cell clusters. Normalized median marker expression is shown. d.p.: in direct proximity to each other **(H)** Absolute CD4 and CD8 cell counts per mm^2^ during treatment. **(I)** T cell phenotypes in EAC patients during treatment were analysed for markers of T cell activation and exhaustion. Fractions of CD4 cells (top row) and CD8 cells (bottom row) were compared among patient groups and visualized by violin plots. **(J)** Ratio of activated and exhausted CD4 cells (top row) and CD8 cells (bottom row) is shown during treatment for REs and NRs. P values in all panels are calculated by the Wilcoxon test, unless stated otherwise.

We assessed the cellular organisation of the immune microenvironment using multiplex imaging mass cytometry (IMC) on 24 regions of interests (ROIs) from 16 NR samples and 42 ROIs from 27 RE samples. We quantified expression of markers for canonical lymphoid and myeloid immune populations (including markers of immune activation, function and regulation), together with tumour cell markers (Fig 6A+B, supplemental fig 6D). First, we explored cellular phenotypes in EAC during neoadjuvant treatment. We extracted single cell phenotype data for 533,734 cells and clustered cells using the phenograph algorithm^39^ which identified 28 distinct cell populations (Fig 6C). Based on molecular expression signatures, we identified phenotypically distinct populations of tumour cells (C1-2, C4-6, C12, C15-C18, C20, C23, C26), immune cells (C3, C7, C9, C11, C14, C19, C22, C24, C27-28) and other cell types (C8, C10, C13, C21, C25)(Fig 6D). CD8^+^ T-cells were common in C14 and C19, and CD4^+^ T-cells in C7, C9, C11 and C14, and macrophages in C3 and C24 (Fig 6D).

To understand phenotypic changes in the tumour and immune landscape in REs and NRs through treatment, we assessed changes in the abundance of different cell populations during treatment (Fig 6E, supplemental fig 6E). Dynamics in NRs during treatment were dominated by a decrease in tumour cell populations (C1-2, C4-6), decreases in the CD4^+^ cell population (C7) and an increase in one CD8^+^ cell population (C19) (Fig 6E). REs also showed decreasing abundances of tumour cell populations (C1, C4, C6, C12, C16, C17) and increases in a population of CD8^+^ cell (C19-cluster) (Fig 6E). To further explore qualitative differences of the immune cell compartment we selected all leukocytes (CD45^+^ cells) and reclustered using immune cell markers of cell activation, exhaustion, antigen-presentation and proliferation. Our immune cell clustering identified 24 distinct leukocyte subpopulations (Fig 6F+G). We observed significant decrease in one activated CD8 cluster (IC15) in REs and NRs and significant decreases in two exhausted CD8 clusters (IC7 and IC18) in NRs, whereby NRs started with higher abundance of exhausted CD8 cells (IC18) in pretreatment samples than REs (Supplemental fig 6F+G).

Given their relevance to immunotherapy, we explored the dynamics of the CD8^+^ and CD4^+^ cells in more detail. NRs showed stable CD8^+^ counts during treatment and a significant decrease in gated CD4^+^ cell counts, whereas the abundance of CD4^+^ and CD8^+^ cells stayed stable in REs (Fig 6H). Correlation of IMC data and quantitative RNA-deconvolution of CD8^+^ and CD4^+^ cells showed a moderate correlation (R_CD8_= 0.42 and R_CD4_= 0.38, supplemental fig 6H). As we noted a marked heterogeneity in the CD4 and CD8 abundance between ROIs in samples where multiple ROIs were analysed (supplemental fig 6I), we assume that the moderate correlation between RNA-Seq and IMC data at least partially resulted from comparing IMC data from small 1-2mm^2^ large ROIs to RNA-derived immune deconvolution from entire tumour sections.

To understand the degree of activation and effector function of the adaptive immune system, we tested CD4^+^ and CD8^+^ cells for expression of molecules informative about T-cell differentiation, regulation and immune activation. We observed a strong decrease in CD4^+^ and CD8^+^ cell fractions expressing CD45RO in NRs, indicative of a loss of non-naive T cells during RCTx (Fig 6I), whereas in REs the abundance of non-naïve CD45RO^+^CD8^+^ and CD45RO^+^CD4^+^ cells was stable (Fig 6I). NRs showed a significant drop in CD8^+^ fractions expressing Granzyme B and in CD4^+^ fraction expressing Granzyme B or HLA-DR (Fig 6I), which indicates a decrease in the highly activated effector T cell phenotype in NRs. In contrast REs showed a decrease in Granzyme B and HLA-DR expression in CD4^+^ cells, whereas CD8^+^ cells stayed in their activated phenotype state during treatment (Fig 6I). The fraction of exhausted CD8^+^ cells and CD4^+^ cells (expressing PD1 or TIM-3) showed significant drops during RCTx in NRs (Fig 6I, supplemental fig 7A). When calculating the ratio between CD8 and CD4 cell counts expressing cytotoxic Granzyme B^+^ and expressing the exhaustion marker PD1^+^, we observed a higher ratio in RE than in NR for both at all timepoints during treatment (Fig. 6J), indicative of a T-cell phenotype with more cytotoxic activity in REs than in NRs, which might explain the better treatment response. Moreover, patients with poor remission (Becker grade 3) showed a significant drop in the CD8^+^Granzyme B^+^/CD8^+^PD1^+^ ratio during treatment, indicative of progressive CD8 cell exhaustion, which was not present in patients with good remission (supplemental fig 7B).

In summary, our IMC data indicates a loss of CD4^+^ and CD8^+^ effectiveness in NRs during treatment via quantitative downregulation and decrease of activated CD4^+^ and CD8^+^ T cells. Compared to NRs, REs had no decrease in T-cell quantity and showed a more activated CD8^+^ and CD4^+^ phenotype at all timepoints during treatment. Our findings strongly suggest that both the quantitative and qualitative composition of the T-cell compartment might represent a central mediator and predictor in treatment response of EAC. When considered together with our genetic and gene expression analyses, we hypothesize that immune escape and a less activated T-cell phenotype in NRs allows tumour cells to resist treatment and could potentially be exploited with new treatment strategies.

### Dynamics of the T-cell Repertoire

Antigen-specific T cell responses are a central feature of the antitumoral response. The TCR repertoire, measurable by TCR sequencing , provides a way to assess the magnitude of the T cell immune response. We therefore analysed the intra-tumoral TCR repertoire in a subset of 54 samples from 9 patients, to evaluate TCR dynamics during neoadjuvant treatment in EAC.

**Figure 7:**
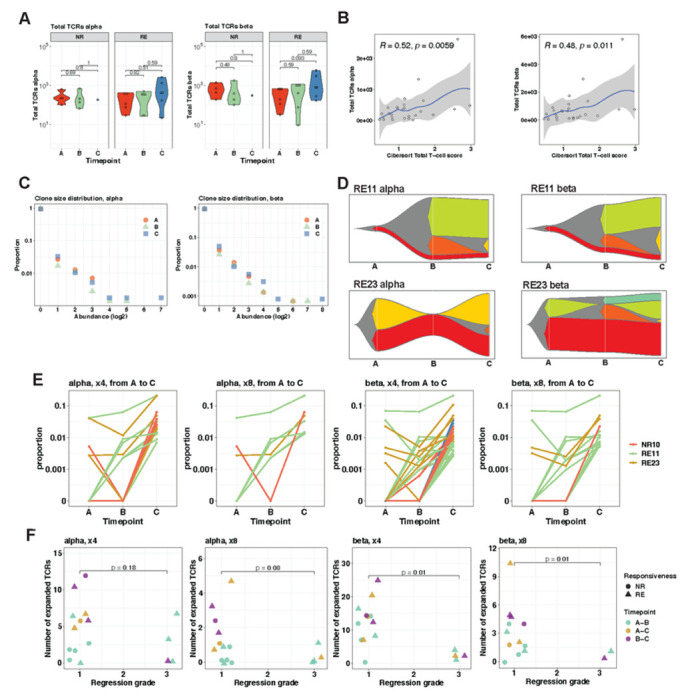
T-cells show clonal expansion in patients with neoadjuvant treatment response. **(A)** Numbers of total TCRs α-chain (left) and β-chain (right) in Responders and Non-Responders during treatment. P values are calculated by the Wilcoxon test. **(B)** Correlation between TCR countsα-chain (left) and β-chain (right) and quantitative deconvolution of T-cells from RNA-Seq data via CIBERSORT. Pearson’s correlation coefficients are reported in the plots **(C)** Abundance distribution profile of TCRs at timepoints A-C. The y-axis shows the proportion of the TCRs which are found at the abundance indicated by the x-axis. **(D)** Fishplots show the occurrence of expanded TCRs at each timepoint for RE11 (top) and RE23 (bottom). **(E)** The proportions of T cell clones that are expanded **≥** 4-fold and ≥8-fold between timepoint A and C. **(F)** Numbers of TCRs expanded ≥4-fold and ≥8-fold during treatment periods (A-B, A-C, B-C), stratified by pathological regression grades. P values are calculated by the Mann-Whitney U test.

We observed an average of 296 total alpha TCR counts and 668 total beta TCR counts in treatment naive NRs and an average of 229 total alpha TCR counts and 380 total beta TCR counts in treatment naive REs, with a slight (non-significant) increase in total TCRs and unique TCRs at the end of neoadjuvant treatment (Fig 7A, supplemental fig 7C). The number of detected TCRs moderately correlated with the absolute score of T-cells from our Cibersort immune cell deconvolution from RNA-Sequencing data (R_alpha_=0.52, R_beta_= 0.48) (Fig 7B).

We examined changes in the proportions of TCR clone sizes in the repertoire during treatment exposure. We did not observe any differences in the overall shape of TCRs clone size distribution between different timepoints (Fig 7C). In order to track individual TCR clones in our longitudinally collected samples, we focussed on TCR clones that had significantly expanded between two timepoints. We observed early (starting between Timepoint A and B) and persisting TCR expansions in both REs with good pathological regression (RE11, RE23), while NR10 and RE5 with poor response to CTx showed late (starting between Timepoint B and C) and intermittent expansions (Fig 7D, supplemental fig 7D). We then considered different thresholds of expansions, which were reactive to treatment and persisting through treatment (=expansions between timepoint A and C) in patients with data from all three timepoints. The trend was observed across the different expansion thresholds, with TCRs that expanded at least 4 or at least 8 times throughout treatment (Fig 7E). Next, we tracked T-cell clone expansions during treatment in patients with different pathological regression grades. Patients with excellent pathological treatment response (Becker grade 1), showed significantly higher numbers of TCRs that expanded at least 4 or at least 8 times between two time points during treatment than patients with poor regression (Fig 7F). In summary our TCR data are suggestive that expansions of individual T-cell clones during treatment play a role in successful treatment response.

Together, we observed a stronger immune cell response of the adaptive immune system with a persisting activated CD8 phenotype and TCR expansions during treatment in patients with good treatment response, which is further supported by the previously observed reduced immune escape in those patients compared to patients with poor treatment response.

## Discussion

Despite advances in treatment, the prognosis for patients with EAC remains poor, with frequent development of resistance to therapy. Genetic analyses of pre- and posttreatment samples from EAC patients have recently started to shed light on tumour clonal dynamics to understand treatment resistance from an evolutionary perspective^4,5,6^. In our prospectively-designed multi-timepoint and multi-omic study, based on longitudinal sampling with pre, during and post neoadjuvant treatment samples from the MEMORI trial, we integrated genetic analyses with transcriptomic analyses and analyses of the tumour immune microenvironment, in order to generate a holistic understanding of treatment resistance.

Somewhat surprisingly, we did not observe clonal replacement for particular subclones during neoadjuvant treatment. Our results are in line previous findings in a multi-region WES study of pre-and posttreatment EAC samples, which similarly reported clonal persistence with rarely new occurring putative drivers posttreatment^5^. We note that another study by Findlay et al did report loss of mutations (and clones) through treatment^4^. Potentially the discrepancy could be explained by low cellularity in some of Findlay et al’s post treatment samples.

Recent evidence implicates a key role of non-mutational resistance mechanisms (where cells can plastically switch phenotype without underlying genetic change) in drug resistance^8,9,40,41,42^. Here, we observed marked changes in transcriptional programmes without clonal replacement during neoadjuvant treatment, which may be evidence of tumour cell plasticity. Although, we emphasise that lots of mutations were gained and “lost” through the time course, so our data and analyses do not definitively rule out that such phenotypic change is a consequence of genetic changes with underlying polyclonal evolution. Nevertheless, the enrichment of epithelial-mesenchymal transition (EMT) gene expression programmes in our post-treatment samples is consistent with previously reported plasticity of these programmes^41,8,43,44^. Further, EMT is known to be controlled by multiple signalling pathways, such as transforming growth factor-β (*TGF-β*), wingless/integrated (*WNT*), *Notch*, and Ras-mitogen-activated protein kinase *(Ras-MAPK)* pathways^41,45,46^, which all showed a significant increase during treatment in EAC. We speculate that inhibition of the pathways that promote EMT, with the goal of maintaining cancer cells in a state susceptible to chemotherapy, could represent a novel avenue for treatment.

Neoantigens and their generated antitumor immune response play a major role in tumour evolution and high neoantigen burden is associated with good prognosis in patients with a variety of cancers^47–49^ and with successful immune-checkpoint therapy^50–53^. Indeed, recently approved combined immuno-chemotherapy for treatment of advanced EAC patients showed a significant improvement in overall survival compared to chemotherapy alone^54,55^. Here, our longitudinal analyses revealed relatively stable neoantigen burdens through treatment, pointing to a lack of stringent immunoediting and consistent with our detection of genetic disruption to antigen presentation machinery. Further, cellular analysis showed remodelling of the T-cell compartment during treatment, with expansion of T-cell clones in responders, and increasing T-cell exhaustion and PDL1 expression in non-responders. Previous studies have shown the immunomodulatory effects of C(R)Tx with an increase in PD-L1 from 45% in pre-NAT EAC to 77% post-CRT^56^. Adjuvant treatment with Nivolumab (anti-PD1 antibody, the T-cell expressed receptor of PDL1) following neoadjuvant RCTx and surgical resection in lymph node positive disease, has been shown significantly increased disease-free survival (DFS) compared to placebo^57^ and results from clinical trials investigating combined checkpoint-blockade and chemotherapy in the perioperative setting are awaited soon^12^. Our data now shed light on immune cell dynamics during chemotherapy and RCTx in EAC and indicate that patients with neoadjuvant chemotherapy and poor pathological response, could potentially benefit from boosting adaptive immunity through combination of chemotherapy and immune checkpoint blockade in the perioperative setting.

In conclusion, our longitudinal multi-omic study shows limited evidence of clonal replacement during neoadjuvant treatment in EAC. Neoadjuvant treatment causes potent modulation of the immune cell compartment, suggesting combination immune-chemotherapy treatments as a potential efficacious approach for neoadjuvant treatment.

## Materials And Methods

### 1. Prospective sample collection within clinical MEMORI trial:

EAC specimens were obtained from the prospective clinical MEMORI trial^13^. Patients with locally advanced EAC and intense FDG tracer uptake of the tumour at baseline FDG-PET-CT ( [ 18F ] - FDG uptake in the tumor at baseline > 1.35 x liver SUV + 2 x standard deviation of the liver SUV) and thus suitable for monitoring and early response prediction by FDG - PET were eligible for MEMORI trial. All patients in the MEMORI trial received 1 cycle of platin-based chemotherapy. 23 patients received FOLFOX (oxaliplatin-5FU) and 5 patients received EOX (Epirubicin, Oxaliplatin, Capecitabine) for their first cycle of neoadjuvant chemotherapy. Metabolic tumour response was assessed with a repeated PET-CT at day 14-21. Responders (REs) were defined as ≥ 35% decrease in maximal standardized uptake values (SUVmax) from baseline and were continued on 3 more cycles of FOLFOX (d14) or 4 more cycles of EOX if EOX was given at 1^st^ cycle. Patients with poor response (< 35% decrease in SUVmax from baseline) were classified as Non-Responder (NRs) and were switched to intensified RCTx (41.4 Gy/23 fractions with weekly carboplatin/paclitaxel) according to the CROSS protocol^58^. Following completion of neoadjuvant treatment, restaging was performed. In the absence of progression to metastatic/unresectable disease, patients underwent surgical resection. 10 NRs and 17 REs were enrolled for longitudinal molecular analyses. Most patients provided 3 cancer samples with one pre-treatment biopsy (Timepoint A), one biopsy collected after the first cycle of chemotherapy (Timepoint B) and the surgical specimen sample after completion of neoadjuvant treatment (either CTx or RCTx) (Timepoint C) (Fig 1B and C). Two patients (RE10 and RE11) had multi-timepoint and multi-region samples (≥ 6 samples per patient). Further clinical and histopathological information is provided in Supplemental table 1. Tumour tissue was fixed and stabilized with the PAX-gene fixation method (PAXgene Tissue FIX Container (Qiagen)), which showed improved preservation biomolecules compared to formalin-fixed tissue^59,60^. For eight samples PAXgene fixated tissue was not available and formalin-fixed tissue was used (supplemental table 8). Blood for germline control was collected in PAXgene Blood DNA Tubes (Qiagen). All patients gave informed consent for collection and molecular analysis of their sample material within the MEMORI trial protocol.

### 2. Section preparation:

PAXgene-fixed paraffin-embedded (PFPE) or formalin-fixed paraffin-embedded (FFPE) tissue was cut into consecutive sections. The first section (4μm) was used for hematoxylin and eosin (H&E) stain. Eight consecutive sections were cut at 10μm under RNAse free conditions onto PEN Membrane Glass Slides (ThermoFisher) for subsequent isolation of DNA and RNA. Then, four consecutive sections were cut at 4μm onto standard slides for IMC analysis and the last section of 4μm was stained with H&E. Both H&E stainings were reviewed by a board-certified pathologist to assure high tissue quality and tumour cellularity. If tumour cellularity was estimated <50%, tumour areas were annotated for later tumour cell enrichment via laser capture dissecting microscopy. Pathological regression in posttreatment sample was assessed based on the tumour regression grades according to Becker^61^. Sections for DNA extractions were stored at −20°C and section for RNA extractions at −80°C, if not processed immediately.

#### 2.1 DNA and RNA isolation:

To increase cellularity and sequencing yield, tissue slides for DNA extraction were stained with methylgreen and tumour cells were microdissected under a laser capture dissecting microscope (PALM MicroBeam, Carl Zeiss Microscopy). From each tube containing the enriched tumour cells, DNA was extracted using the PAXgene Tissue DNA Kit (Qiagen) for PFPE tissue and the High Pure FFPET DNA Isolation Kit (Roche Molecular Systems, Basel, Switzerland) for FFPE tissue according to the manufacturer’s instructions. Matching Blood-DNA was extracted with the QIAamp DNA Blood Mini Kit (Qiagen). The integrity of DNA molecules was assessed via D5000 Tapestation Assay (Life Technologies, Paisley, UK). Samples with a total DNA yield higher than 10ng and DNA peak detection at > 1000bp were taken forward for WES library preparation.

RNA extractions from PFPE sections were performed using the PAXgeneTissue RNA Kit from Qiagen according to the manufacturer’s protocol. For FFPE sections RNA was extracted using the High Pure FFPET RNA Isolation Kit (Roche Molecular Systems, Basel, Switzerland). RNA quantity was measured using the Qubit fluorometer RNA High Sensitivity assay (Life Technologies, Paisley, UK). Quality control was performed using the High Sensitivity RNA TapeStation system (Agilent, Santa Clara, California, USA). RNA quality was assessed using the DV200 value. RNA samples with a DV200 > 50% were eligible for further analysis.

#### 2.2 Preparation of whole exome sequencing libraries:

Libraries were prepared using the SureSelectXT Low Input Target Enrichment System with Dual Indexing (Agilent Technologies, Santa Clara, US) according to manufacturer’s instructions. A short mechanic fragmentation step using a Covaris S2 Sonicator to generate 150-200bp long fragments was performed and 8 to 14 PCR cycles were used for library enrichment depending on the DNA input. After purification, libraries were quantified by Qubit and run on the Agilent Tapestation using HSD1000 screentapes. Samples with sufficient library DNA yield and characteristic fragment size distribution (~200-500bp) were further subjected to deep (~300x depth) WES.

#### 2.3 Preparation of RNA sequencing libraries:

Libraries were prepared using the QuantSeq 3’mRNASeq Library Prep Kit FWD (Lexogen GmbH), according to the manufacturer’s protocol with minor modifications. To increase the total RNA library yields, the pre-PCR incubation time was increased to 1h. After purification, libraries were quantified by Qubit and run on the Agilent Tapestation using D1000 screentapes. Samples with sufficient library cDNA yield and characteristic fragment size distribution (~170-700bp) were further subjected to RNA-Sequencing.

#### 2.4 WES and RNA Sequencing:

Sequencing libraries were multiplexed and sequenced on an Illumina Novaseq, typically using S2 flow cells. Read length and depth was varied as required by library composition. Target depth for WES was 250x and >2 million reads per sample for RNA-Seq. Sequencing was performed by the Genome Centre at Queen Mary University London.

### 3. Bioinformatical analysis of Whole-exome sequencing data

#### 3.1 Whole-exome sequencing – alignment:

Contaminating adapter sequences were removed using Skewer v0.2.2^62^, with a maximum error rate of 0.1, minimum mean quality value of 10 and a minimum read length of 50 after trimming using options “-I 50 -r 0.1 -d 0.03 -Q 10 -n”. The trimmed and filtered reads from each sequencing run and library where separately aligned to the GRCh38 reference assembly of the human genome^63^ using the BWA-MEM algorithm v0.7.17^64^. Following the GATK bestpractices and the associated set of tools v4.1.4.1^65–67^ reads were sorted by coordinates (GATK SortSam), independent sequencing runs from the same tissue sample were merged and duplicated reads were marked using GATKs MarkDuplicates. The coverage statistics of the final bam files were verified with Qualimap v2.2.1^68^.

#### 3.2 Copy number analysis:

Copy number analysis and cellularity estimates were performed with Sequenza^69^. Pre-processing of bam files to seqz files was done using the sequenza-utils bam2seqz algorithm and binned using the sequenza-utils seqz_binning -w50 command. The per patient set of break points, binned depth-ratio and B-allele frequencies data from binned seqz files were then inputted into the sequenza algorithm (version 2.1.2) to determine allele specific copy-numbers, ploidy Ψ and purity ρ estimates^69^. The threshold segment length for fitting cellularity and ploidy parameters was set to 10^6^ bp to filter out short segments, that can cause noise for cellularity and ploidy estimates. The initial parameter space searched was restricted to [ρ | 0.1 ≤ ρ ≤1] and [Ψ | 1 ≤ Ψ ≤ 5]. Upon manual review of the results, we identified several samples with unreasonable fits (cases where calls suggested extremely variable ploidy values across samples from the same patient). For these samples, we manually identified alternative solutions consistent with the other samples and somatic variant calls. Clonal CNAs were defined as CNAs present in all samples of a patient, subclonal CNAs were defined as CNAs present in > 1/3 of samples, but not all samples of a patient and private CNAs as CNAs present in ≤ 1/3 of samples of a patient. For patients with only 2 available samples subclonal/private CNAs were defined as CNAs present in 1 sample. To calculate the fraction of exome, that changes its CNS between timepoint A and B (Fig 3D), 15 patients with available samples from timepoint A and B and no multi-region samples at timepoint A or B were included.

#### 3.3 SNV detection:

Somatic mutations were first called for each tumour sample separately against matched blood samples with Mutect2 (version 4.1.2.0)^70^. Variants detected were annotated using ANNOVAR^71^. Variants detected in any tumour sample (marked PASS, coverage AD 5 in both normal and tumour, at least 3 variant reads in the tumour and a maximum variant read of 0 in the normal reference genotype) were merged into a single list of “candidate mutations”. For later MOBSTER-analysis^16^ for multi-region/multi-timepoint samples (see below), SNV caller bam-readcount^72^ was used to call nucleobases and coverage at each candidate mutation position in all samples of a respective patient. Candidate driver genes of EAC were annotated using 108 cancer driver genes for oesophageal and gastric cancer reported by IntOGen^®73^.

#### 3.4 Identification of subclonal and clonal mutations per sample:

For each sample “candidate mutations” were classified as clonal or subclonal mutation using MOBSTER^74^. Based on the calculated variant allele frequency (VAF), i.e., the ratio of read counts mapping to the mutant allele, over the total coverage at the variant locus, which was previously normalized by the tumour purity and tumour copy number segments, MOBSTER clusters input mutations as clonal peak mutations (=clonal mutations) or tail mutations for neutral mutations (= subclonal mutations).

#### 3.5 Construction of phylogenetic trees:

For phylogenetic tree analysis we analysed SNVs from multi-region/multi-timepoint samples of each patient using MOBSTER for multiple samples. To avoid previously described biases in clonality analysis of multi-region/multi-timepoint analysis^16^, MOBSTER excludes mutations belonging to the evolutionary neutral-tail for clonality analysis in multi-region/multi-timepoint samples. From non-tail mutations maximum-parsimony trees were reconstructed with the Parsimony Ratchet method^75^ using the phangorn package for R^76^. Included non-tail mutations within a sample were considered to be mutated (state 1) and others considered to be non-mutated (state 0) in a given sample. The Ratchet was run for a minimum of 10 and a maximum of 10^3^ iterations and terminated after 10 rounds without improvement. The acctran algorithm^76–78^ was utilized to approximate ancestral character states. From these a set of shared mutations between samples and mutations that were uniquely mutated on each clade of the phylogeny were obtained.

#### 3.6 Extraction of mutational signatures and dN/dS analyses:

The extraction of mutational signatures in tumour samples was carried out with SigProfiler^79,80^, using COSMIC signatures as reference signatures and the default settings. Unfiltered signatures for each tumour sample are shown in the extended data (supplemental fig 1C). In order to reduce the number of called mutation signatures to more prevalent ones, we rerun the analysis excluding signatures that had a COSMIC signature weight of <5% in any of the responsiveness groups at a given timepoint. Next, we evaluated mutational footprints of chemo- and radiochemotherapy in our cohort. COSMIC signatures were called separately for treatment-naive mutations, mutations occurring under chemotherapy (= “CTx-SNVs”) and mutations occurring under RCTx (=“RCTx-SNVs ”). Treatment-naive mutations included mutations at timepoint A in REs and NRs, CTx-SNVs included newly occurring mutations at timepoint B in REs and NRs and newly occurring mutations at timepoint C in REs and RCTx-SNVs harboured newly occurring mutations at timepoint C in NRs. dN/dS ratios were calculated for treatment-naïve mutations, CTx-SNVs and RCTx-SNVs using the dNdScv R package^81^.

#### 3.7 Genetic alterations and RNA expression in EAC drivers:

SNVs, indels and CNA were assessed in 108 IntOGen^®^ drivers^17^ of oesophageal and gastric cancer. To assess the number of altered driver genes in each sample, driver genes were counted as altered if they harboured one or more SNVs, indels or CNAs. To assess the correlation between CNS and RNA expression of 15 high frequency driver genes, samples with matching WES and RNA-Sequencing data were analysed (n=66). RNA-Sequencing data was pre-processed as described in 4.1 and 4.2. Changes in expression levels of driver genes between treatment-naive samples and post-treatment samples were calculated by Wilcoxon tests. For correlation analyses between the expression level and the CNS of driver genes, the expression level was adjusted for the estimated tumour cellularity. Correlation between expression levels and CNS were calculated for each driver genes by Pearson correlation. For expression analyses of driver genes in samples with mutated and non-mutated driver status, only CDKN2A and TP53 displayed a sufficiently powered analysis. The expression level of the respective driver was adjusted for the estimated tumour cellularity. Expression levels between mutated and non-mutated samples were compared by the Wilcoxon test.

#### 3.8 HLA haplotyping:

HLA haplotyping for each patient was carried out with the raw paired fastqs of blood-derived normal samples using Optitype^82^. The pair of A, B and C alleles with the highest score were taken as a patient’s haplotype.

#### 3.9 Calling Immune escape:

We predicted four types of immune escape mechanisms: (1) somatic mutations in one of the HLA alleles or (2) in the *B2M* gene^83^; (3) loss of an HLA haplotype through LOH^84^; and (4) PD-L1 overexpression^85^.

Somatic mutations in the HLA locus were called using polysolver^83^. First, alleles were converted into a polysolver-compatible format (lower case, digits separated by underscore) and outputted into a patient-specific winners.hla.txt file, following the standard output of polysolver. Next, the mutation detection script of polysolver (shell_call_hla_mutations_from_type) was run on matched tumour- normal pairs to call tumour-specific alterations in HLA-aligned sequencing reads using MuTect (v1.16). In addition, Strelka2 (v2.9.9)^86^ was run independently to detect short insertions and deletions in HLA-aligned reads as this software offers increased sensitivity over polysolver’s default caller. Finally, both single nucleotide mutations and insertions/deletions passing quality control were annotated by polysolver’s built-in annotation script, shell_annotate_hla_mutations. LOH at the HLA locus was assessed using the software LOHHLA^84^ and sequenza^69^. First, the allele-specific copy number as predicted by sequenza at the HLA-A, -B, -C loci was evaluated. Samples with a predicted minor allele copy number of 0 (e.g. 2:0, 3:0) were labelled as candidate LOH. Then, we ran LOHHLA with the previously generated winners.hla.txt as input. A type-I allele of a patient was annotated as “allelic imbalance” (AI) if the p-value testing the difference in evidence for the two alleles was lower than 0.01. Alleles with AI were labelled as LOH if the following criteria held: (i) the predicted copy number of the lost allele was below 0.5 with confidence interval strictly below 0.7; (ii) the copy number of the kept allele was above 0.75; (iii) the number of mismatched sites between alleles was above 10. All LOHHLA-based LOH called were in candidate LOH samples, but some candidate LOH could not be confirmed most probably due to low purity and read density.

Mutations in *B2M* were called if the sample contained contained a nonsynonymous somatic, frameshift (FS), stop-loss or stop-gain mutation located inside the exonic regions of the exons of the *B2M* gene, as called by ANNOVAR^71^. PD-L1 overexpression was assessed using matched RNA-seq data from the samples. RNA-Seq raw data was pre-processed and then normalized as described in 4.1 and 4.2. The mean expression value of normalized gene counts (in transcripts per million mapped reads (TPM)) of PD-L1) in treatment-naive EAC samples was calculated and considered as PDL-1 treatment-naive baseline. PDL-1 overexpression was defined as ≥ PD-L1 treatment-naive baseline + 1 standard deviation.

#### 3.10 Calling neoantigens:

Neoantigens were predicted from variant call tables and HLA types using the NeoPredPipe^87^, python-based pipeline combining Annovar and netMHCpan 4.0. Briefly, all quality-controlled somatic mutations were annotated using Annovar^71^, and for all non-synonymous exonic mutations the mutated peptide sequence was predicted. We took any 9- and 10-mer spanning the mutated amino acid(s), resulting in either (i) a 19-aa window for SNVs or (ii) a peptide until the next predicted stop codon for FSs. These peptides were evaluated according to their novelty and predicted binding strength to the patient’s six-allele HLA set. Peptides novel compared to the healthy human proteome with binding rank 2 and below (amongst the best 2% of binders compared to a large set of random peptides) were reported as neoantigens. All patient-specific HLA alleles were used for neoantigen predictions, regardless of mutation or LOH status of the HLA locus. We considered a mutation neoantigenic if at least one of its downstream mutated peptides were a neoantigen with respect to any of the patient’s six HLA alleles. The clonality of neoantigentic mutations was assessed based on MOBSTER^74^ analyses (more details in 3.4). To calculate the enrichment/depletion of neoantigens in regions of different copy number states, we calculated for each sample, the ratio between the fraction of copy-number adjusted NeoSNVs present in a certain CN category (loss, cnLOH, high LOH, diploid, gain and amplification) and the fraction of genome showing the respective CN category.

#### 3.11 Expression of neoantigens:

To assess the expression levels of neoantigenic peptides, we analyzed gene expression levels of genes harbouring neoantigenic mutations. RNA-Seq raw data was pre-processed and then normalized as described in 4.1 and 4.2. As a proxy for neoantigen expression, expression levels (CPM) of protein-coding genes harbouring neoantigenic mutations were assessed. Mean expression levels of neoantigens were calculated for each sample and compared between samples from different timepoints via Wilcoxon tests. For analyses of neoantigen expression levels in low and high CD4 and CD8 cell groups, CD4 and CD8 T-cell levels were deconvoluted from normalized RNA-sequencing data using CIBERSORT^30^ (more details in methods part 4.5). Samples were assigned to high and low CD4 and CD8 cell groups based on the median CD4 and CD8 score of the study sample.

### 4. Bioinformatic analysis of RNA-Sequencing

#### 4.1 RNA sequencing – alignment and filtering of RNA samples:

After initial quality control with FastQC^88^ and default adaptor trimming with Skewer^62^, paired-end reads were aligned to GRCh38 reference genome and version 28 of the Gencode GTF annotation using the STAR 2-pass method^89^. Read groups were added with Picard v.2.5.0^90^. Per gene read counts were produced with htseq-count that is incorporated into the STAR pipeline^89^. Raw gene counts were first filtered for reads uniquely assigned to non-ribosomal protein-coding genes located on canonical chromosomes (chr1-22, X and Y). If samples had less than 1.5M of these ‘usable’ reads they were re-sequenced to improve coverage. Where possible, the same library preparation pool was sent again for sequencing. These

$$⊤–ups^{’}proved\to betruetechnicalreplicates,\sin\;cetheres\underline{t}\in \;g\geq \;\neq \;\exp\;ressionofthere–sequencedsamp\;\leq \;sclusteredveryclosel$$

top-ups’ of the second library where necessary) contained <1.5x10^6^ reads, samples were excluded for further downstream analysis.

#### 4.2 Gene expression normalisation and filtering of expressed genes:

A master table including raw read counts for each samples of 58,243 genes were converted to a DGEList object via DESeq2^91^. Next, a list of expressed genes (n=11,900) was determined by edgeR^92^ default filtering, to determine genes with sufficiently large counts to be retained in a statistical analysis. Normalization and conversion into counts per million (CPM) was performed using edgeR^92^. Samples from different sequencing batches and post-treatment samples with different fixation methods clustered mixed on a PCA, indicating no notable batch effects (Supplemental fig. 8A+B).

#### 4.3 Pathway enrichment clustering:

Hallmark pathways were download from MSigDB^28^ and unrelated pathways (SPERMATOGENSIS, MYOGENESIS and ANDROGEN_RESPONSE) were excluded, leading to a final list of 47 pathways. Based on their biological function pathways were categorized into 4 groups: oncogenic, immune, cellular stress, stromal and other. For each tumour sample, the CPM expression of expressed genes converted to entrez gene IDs (n= 10,463) was used as input for single sample gene set enrichment analysis using the GSVA R package^93^. The mean and standard deviation of enrichment was then recorded for each sample. Unsupervised clustering of samples based on gene set enrichment scores for each pathway was used to cluster tumour samples via principal component analysis. Loadings of defined hallmark pathways and hallmark classes (oncogenic, immune, stromal, stress) on the PCA dimensions of interest was visualized separately. Pathways categorised in the class of “others” and regarded as cancer unrelated were removed from the loading plot for clearer visualisation purposes. As PCA clustering showed most samples from timepoint A and B clustering separately from timepoint C on PC1, differentially expressed pathways between samples from timepoint A/B and timepoint C were determined via *FDR adjusted p-values determined via* Wilcoxon signed-rank tests. Supervised clustering with significantly differentially expressed hallmark pathways between samples from timepoint A/B and timepoint C were visualised in a heatmap created with the pheatmap R package.

#### 4.4 KEGG pathway enrichment analysis:

For each tumour sample DESeq normalized counts (or mean normalized counts across samples for multi-region sampled tumours) were used for KEGG pathway enrichment analysis. For each gene, log2 fold changes between patient groups were calculated via DESeq analysis to determine differential expression. Conversion to entrez gene IDs and gene symbols was performed using the Biological Id Translator function from ClusterProfiler (v4.2.2)^94^. The enrichment of KEGG pathways for differentially expressed genes between patient groups was determined with enrichKEGG from ClusterProfiler^94^. Adjusted p-values were calculated using the false discovery rate. Enriched pathways with adjusted p-value <0.1 were plotted in Fig 4D and significantly enriched pathways (p<0.05) were demarcated by a dotted line in the graph.

#### 4.5 Immune deconvolution from RNA-Sequencing data

Immune cells were deconvoluted with CIBERSORT^30^ a computational method for quantifying cell fractions from bulk tissue gene expression profiles based on support vector regression with prior knowledge of expression profiles from purified leukocyte subsets. For each tumour sample, the CPM expression of genes converted to Gene Symbols was used as input for CIBERSORT single sample immune deconvolution and was uploaded to the online available CIBERSORT tool (https://cibersort.stanford.edu/index.php). A LM22 signature file compromising 22 immune cell types and included in the deconvolution tool was used for deconvolution. Deconvoluted immune cell data from our samples was outputted as absolute and relative scores. For further analysis CIBERSORT output tables were processed in R. For each sample CIBERSORT scores of “Macrophages.M0”, “Macrophages.M1” and “Macrophages.M2”, were summed to a joint score for “Macrophages”, scores of activated and resting cells of the same cell types were summed (e.g. “NK.cells.resting” and “NK.cells.activated” were summed to a “NK cell” score), scores for “B.cells.naive”, “B.cells.memory” and “Plasma.cells” were summed to “Other B cells”, scores for “T.cells.follicular.helper”, “T.cells.regulatory..Tregs.”, “T.cells.gamma.delta” were joint to “Other T cells” and scores for “T.cells.CD4.naive”, “T.cells.CD4.memory.activated” and “T.cells.CD4.memory resting” were added to common score for “CD4 T-cells”. For validation purposes further immune cell deconvolution methods were used. For each tumour sample, the CPM expression of protein-coding genes and expressed genes converted to entrez gene IDs (n= 10,463) was used as input for single sample gene set enrichment analysis using the Consensus^TME^ gene sets^31^ and the GSVA R package^93^ in R. As a third immune deconvolution tool Syllogist^32^ was used. For each tumour sample, the CPM expression of protein-coding genes converted to HUGO nomenclature (n= 39,921) was used as input for the provided Syllogist script^32^, which was run in R. Odds ratios of immune cells of interests were visualized in bar graphs generated in R.

##### IMC staining:

IMC stainings were performed on 54 ROIs from PAXgene fixed samples and 12 ROIs from FFPE samples (supplemental table 8). Incubation, rehydration and epitope retrieval was performed on 4μm thick tissue sections as described in previous work^95^. The sections were then stained with a mix of metal-labeled primary antibodies (Supplemental table 4) diluted in TBS with 0.5% BSA and incubated at 4°C overnight. Sections were then rinsed, stained with Iridium Cell-ID and air-dried as previously described^95^. Slides were stored at room temperature until image acquisition.

##### IMC image acquisition

A 1-2 mm2 image per sample was acquired using a Hyperion Imaging System (Fluidigm). Tuning of the instrument was performed according to the manufacturer’s instructions. Regions of interest were determined based on a pathologist’s annotations on consecutive HE-stained section of each sample. Tissue sections were laser-ablated spot-by-spot at 200 Hz resulting in a pixel 2 size/resolution of 1 mm. Preprocessing of the raw data was conducted using the CyTOF software v7.0. Image acquisition control was performed using MCD Viewer v1.0.560.6. For larger samples multiple ROIs were obtained.

##### IMC raw data processing:

Raw data were exported as MCD files. MCD files were converted into tiff files and segmented into single cells using an unbiased, supervised analysis pipeline adapted from https://github.com/BodenmillerGroup/ImcSegmentationPipeline. In more detail, nucleated cells were segmented using Ilastik v1.3.3^96^ and CellProfiler v4.1.3^97^. Using Ilastik, pixels were classified into nuclei, cytoplasm and background. A probability map for the three classifications was generated and exported to create a cell mask with CellProfiler. Data folders containing tiff images of the 21 markers and the combined cell mask were loaded into histoCAT v1.76. In histocat mean marker intensity of pixels and spatial features of segmented cells were analyzed and exported as a table containing single cell data for each marker. Subsequent processing of single-cell level data was performed to OMIQ.

##### Cluster analysis

IMC single-cell level data and clinical data for each sample were uploaded in OMIQ. Arcsinh transformation (factor 0.5) was performed. To exclude batch effects based on PFPE and FFPE fixation methods, mean expression of each marker between FFPE and PFPE post-treatment samples was assessed (supplemental fig 8C). 21 markers with specific staining in the mcd images and no significant difference in staining intensity between post-treatment FFPE and PFPE samples (supplemental fig 8C) were further used for principal component analysis. PCA showed no major batch effect between samples from different fixation methods (supplemental fig 8D, E). The three FFPE ROIs clustering slightly apart in the PCAs (circled in the PCAs) were from the same patient (RE23) (supplemental fig 8D), indicating most likely a different biology in thisn patient. Thus all 21 markers were eligible for further analyses. Visualization of the global single-cell landscape was performed in OMIQ after arcsinh transformation (factor 0.5) using Opt-SNE based on channels (CD163, CD204, CD3, CD45RO, CD45, CD4, CD8a, Ecadherin, Eomes, GzmB, Ki67, PD-1, PD-L1, PanCK, SMA, Tim-3) (random seed 3367, max iterations 1000, opt-SNE end 5000, perplexity 30, theta 0.5 and verbosity 25). Phenograph clusters were visualized using the Phenograph algorithm based on the cell markers (CD163, CD204, CD3, CD45RO, CD4, CD8a, Ecadherin, Eomes, GzmB, Ki67, PD-1, PD-L1, PanCK, SMA, Tim-3) (k nearest neighbours 60, Louvain seed 8216, distance metric euclidean, Louvain runs 1, number of results 1) and Z-score of median marker expression of 28 phenograph clusters was visualized with a heatmap in OMIQ. For initially ambiguous clusters expressing markers of distinct cell types (C1, C4, C6, C11, C14, C18, C23 and C24), individual cluster cells were manually reviewed on the original image file in order to define the cluster’s cell type. Exemplary IMC stainings of cells from those clusters are shown in supplemental fig 9. Based on their marker profiles, all clusters were classified into different cluster types (tumour cell, immune cell and clusters of other cells). For immune cell clustering, CD45^+^ cells were gated based on their CD45 expression. Marker positivity was determined based on thresholds obtained by sampling background areas and positive cells of the image in MCD viewer. Visualization of the immune single-cell landscape was performed in OMIQ after arcsinh transformation (factor 0.5) using Opt-SNE based on channels (CD163, CD204, CD38, CD3, CD45RO, CD4, CD8a, Eomes, FoxP3, GzmB, HLA-DR, Ki67, PD-1, TCF1, Tbet, Tim-3) (random seed 8836, max iterations 1000, opt-SNE end 5000, perplexity 30, theta 0.5 and verbosity 25). Phenograph clusters were visualized using the Phenograph algorithm based on the same markers (k nearest neighbours 50, Louvain seed 7583, distance metric euclidean, Louvain runs 1, number of results 1). Differences in cell counts of identified clusters between REs and NRs at different timepoints were analysed by anova tests (alternative Fig 6E, H) and * indicates p < 0.05, ** p < 0.01 and *** p < 0.001.

##### Phenotype analyses of CD4+ and CD8+ T cells:

CD4^+^ and CD8^+^ cells were gated from each sample based on their CD4 and CD8a expression respectively. Marker positivity was determined based on thresholds obtained by sampling background areas and positive cells of the image in MCD viewer. The total imaging mass cytometry dataset consisted of n = 30,881 CD4^+^ cells and n= 15,468 CD8^+^ cells. Quantification of distinct T-cells with different phenotypes, was assessed by further subsampling CD4^+^ and CD8^+^ cells based on their expression of markers for differentiation, transcriptional programming, activation/exhaustion, and cytotoxicity (CD45RO, Granzyme B, HLA-DR, PD-1, Eomes, Tim-3, CD38, Ki67). Marker positivity was determined based on thresholds obtained by sampling background areas and positive cells of the image in MCD viewer. Gated T-cell composition including counts per sample and fractions of respective parent cell, were exported from OMIQ. Different T-cell phenotypes between patient groups were visualized using violinplots generated with the ggplot2 function in R studio and compared between patient groups by Wilcoxon tests.

##### TCR sequencing:

The α and β chains of the TCR repertoire of 27 samples from 10 patients were sequenced based on total RNA isolated from tumour samples. Briefly, the method introduces unique molecular identifiers attached to individual cDNA molecules to provide a quantitative and reproducible method of library preparation. Full details for both the experimental TCRseq library preparation and the subsequent computational analysis (V, J, and CDR3 annotation) using Decombinator are published in our previous work^98,99^

##### TCR statistical analysis:

Statistical analyses were performed in R. Wilcoxon rank tests were used to compare the abundance of detected TCRs between timepoints in NRs and RE. For correlation analysis between deconvoluted T-cells from Consensus^TME^ and total TCRs, Consensus^TME^ scores for ‘CD4 T-cells’, ‘CD8 T-cells’ and ‘regulatory T-cells’ were summed to a ‘Total T-cell score’ for each sample. Correlation between calculated ‘Total T-cell scores’ and abundance of detected TCRs was calculated using Pearson correlation analysis. To calculate expansions of TCRs during treatment, individual TCRs were normalized in each sample to TCRs/million. The sets of TCRs that had expanded in proportion by at least 4 times, and at least 8 times, between timepoints A and B, A and C, and B and C were identified. The proportions of the TCRs that had expanded between timepoint A and C were tracked across all timepoints and plotted for patients with TCR data from all 3 timepoints. The number of at least 4-fold and 8-fold expanded TCRs between any 2 timepoints was plotted for patients with different pathological regression scores. Statistical comparisons between the number of expanded TCRs and regression scores were calculated by Mann-Whitney U tests.

## References

[1] Sung H. Global cancer statistics 2020: GLOBOCAN estimates of incidence and mortality worldwide for 36 cancers in 185 countries. CA Cancer J Clin, doi:10.3322/caac.21660 (2021). 33538338

[2] Morgan E. International trends in oesophageal cancer survival by histological subtype between 1995 and 2014. Gut 70, 234–242, doi:10.1136/gutjnl-2020-321089 (2021). 32554620

[3] Davies A. R. Tumor stage after neoadjuvant chemotherapy determines survival after surgery for adenocarcinoma of the esophagus and esophagogastric junction. J. Clin. Oncol. 32, 2983–2990, doi:10.1200/JCO.2014.55.9070 (2014). 25071104

[4] Findlay J. M. Differential clonal evolution in oesophageal cancers in response to neo-adjuvant chemotherapy. Nat Commun 7, 11111, doi:10.1038/ncomms11111 (2016). 27045317 PMC4822033

[5] Murugaesu N. Tracking the genomic evolution of esophageal adenocarcinoma through neoadjuvant chemotherapy. Cancer Discov 5, 821–831, doi:10.1158/2159-8290.CD-15-0412 (2015). 26003801 PMC4529488

[6] Noorani A. A comparative analysis of whole genome sequencing of esophageal adenocarcinoma pre- and post-chemotherapy. Genome Res 27, 902–912, doi:10.1101/gr.214296.116 (2017). 28465312 PMC5453324

[7] Marine J. C., Dawson S. J. & Dawson M. A. Non-genetic mechanisms of therapeutic resistance in cancer. Nat Rev Cancer 20, 743–756, doi:10.1038/S41568-020-00302-4 (2020). 33033407

[8] Boumahdi S. & de Sauvage F. J. The great escape: tumour cell plasticity in resistance to targeted therapy. Nat Rev Drug Discov 19, 39–56, doi:10.1038/S41573-019-0044-1 (2020). 31601994

[9] Shaffer S. M. Rare cell variability and drug-induced reprogramming as a mode of cancer drug resistance. Nature 546, 431–435, doi:10.1038/nature22794 (2017). 28607484 PMC5542814

[10] Househam J. Phenotypic plasticity and genetic control in colorectal cancer evolution. Nature 611, 744–753, doi:10.1038/s41586-022-05311-x (2022). 36289336 PMC9684078

[11] Galluzzi L., Buque A., Kepp O., Zitvogel L. & Kroemer G. Immunological Effects of Conventional Chemotherapy and Targeted Anticancer Agents. Cancer Cell 28, 690–714, doi:10.1016/j.ccell.2015.10.012 (2015). 26678337

[12] Bang Y. J. KEYNOTE-585: Phase III study of perioperative chemotherapy with or without pembrolizumab for gastric cancer. Future Oncol 15, 943–952, doi:10.2217/fon-2018-0581 (2019). 30777447

[13] Lorenzen S. PET-directed combined modality therapy for gastroesophageal junction cancer: Results of the multicentre prospective MEMORI trial of the German Cancer Consortium (DKTK). Eur J Cancer 175, 99–106, doi:10.1016/j.ejca.2022.07.027 (2022). 36099671

[14] Ross-Innes C. S. Whole-genome sequencing provides new insights into the clonal architecture of Barrett’s esophagus and esophageal adenocarcinoma. Nat. Genet. 47, 1038–1046, doi:10.1038/ng.3357 (2015). 26192915 PMC4556068

[15] Dulak A. M. Exome and whole-genome sequencing of esophageal adenocarcinoma identifies recurrent driver events and mutational complexity. Nat. Genet. 45, 478–486, doi:10.1038/ng.2591 (2013). 23525077 PMC3678719

[16] Caravagna G. Subclonal reconstruction of tumors by using machine learning and population genetics. Nat Genet 52, 898–907, doi:10.1038/S41588-020-0675-5 (2020). 32879509 PMC7610388

[17] Gonzalez-Perez A. IntOGen-mutations identifies cancer drivers across tumor types. Nat Methods 10, 1081–1082, doi:10.1038/nmeth.2642 (2013). 24037244 PMC5758042

[18] Frankell A. M. The landscape of selection in 551 esophageal adenocarcinomas defines genomic biomarkers forthe clinic. Nat. Genet. 51, 506–516, doi:papers2://publication/uuid/151598F9-66DE-4ADE-84E9-1075C1E80487 (2019). 30718927 10.1038/s41588-018-0331-5 PMC6420087

[19] Weaver J. M. J. Ordering of mutations in preinvasive disease stages of esophageal carcinogenesis. Nat. Genet. 46, 837–843, doi:10.1038/ng.3013 (2014). 24952744 PMC4116294

[20] Nones K. Genomic catastrophes frequently arise in esophageal adenocarcinoma and drive tumorigenesis. Nat Commun 5, 5224, doi:10.1038/ncomms6224 (2014). 25351503 PMC4596003

[21] Pich O. The mutational footprints of cancer therapies. Nat Genet 51, 1732–1740, doi:10.1038/s41588-019-0525-5 (2019). 31740835 PMC6887544

[22] Secrier M. Mutational signatures in esophageal adenocarcinoma define etiological ly distinct subgroups with therapeutic relevance. Nat Genet 48, 1131–1141, doi:10.1038/ng.3659 (2016). 27595477 PMC5957269

[23] Noorani A. Genomic evidence supports a clonal diaspora model for metastases of esophageal adenocarcinoma. Nat Genet 52, 74–83, doi:10.1038/S41588-019-0551-3 (2020). 31907488 PMC7100916

[24] Alexandrov. Signatures of Mutational Processes in Human Cancer, <https://cancer.sanger.ac.uk/signatures/signatures_v2/> (10.1038/nature12477 PMC3776390 23945592

[25] Rose Li Y. Mutational signatures in tumours induced by high and low energy radiation in Trp53 deficient mice. Nat Commun 11, 394, doi:10.1038/S41467-019-14261-4 (2020). 31959748 PMC6971050

[26] Cancer Genome Atlas Research, N. Integrated genomic characterization of oesophageal carcinoma. Nature 541, 169–175, doi:10.1038/nature20805 (2017). 28052061 PMC5651175

[27] Behjati S. Mutational signatures of ionizing radiation in second malignancies. Nat Commun 7, 12605, doi: 10.1038/ncomms12605 (2016). 27615322 PMC5027243

[28] Liberzon A. The Molecular Signatures Database (MSigDB) hallmark gene set collection. Cell Syst 1, 417–425, doi:10.1016/j.cels.2015.12.004 (2015). 26771021 PMC4707969

[29] Jimenez-Sanchez A. Unraveling tumor-immune heterogeneity in advanced ovarian cancer uncovers immunogenic effect of chemotherapy. Nat Genet 52, 582–593, doi:10.1038/s41588-020-0630-5 (2020). 32483290 PMC8353209

[30] Chen B., Khodadoust M. S., Liu C. L., Newman A. M. & Alizadeh A. A. Profiling Tumor Infiltrating Immune Cells with CIBERSORT. Methods Mol Biol 1711, 243–259, doi:10.1007/978-1-4939-7493-1_12 (2018). 29344893 PMC5895181

[31] Jimenez-Sanchez A., Cast O. & Miller M. L. Comprehensive Benchmarking and Integration of Tumor Microenvironment Cell Estimation Methods. Cancer Res 79, 6238–6246, doi:10.1158/0008-5472.CAN-18-3560 (2019). 31641033

[32] Lu I. N. Tumor-associated hematopoietic stem and progenitor cells positively linked to glioblastoma progression. Nat Commun 12, 3895, doi:10.1038/S41467-021-23995-z (2021). 34162860 PMC8222381

[33] Schumacher T. N. & Schreiber R. D. Neoantigens in cancer immunotherapy. Science 348, 69–74, doi:10.1126/science.aaa4971 (2015). 25838375

[34] Lu Y. C. & Robbins P F. Cancer immunotherapy targeting neoantigens. Semin Immunol 28, 22–27, doi:10.1016/j.smim.2015.11.002 (2016). 26653770 PMC4862880

[35] (ed Food and Drug Administration) (June 2021).

[36] Van den Eynden J., Jimenez-Sanchez A., Miller M. L. & Larsson E. Lack of detectable neoantigen depletion signals in the untreated cancer genome. Nat Genet 51, 1741–1748, doi:10.1038/s41588-019-0532-6 (2019). 31768072 PMC6887557

[37] Rooney M. S., Shukla S. A., Wu C. J., Getz G. & Hacohen N. Molecular and genetic properties of tumors associated with local immune cytolytic activity. Cell 160, 48–61, doi:10.1016/j.cell.2014.12.033 (2015). 25594174 PMC4856474

[38] Eszter Lakatos M. J. W., Schenck Ryan O., Cross William C. H., Househam Jacob, Werner Benjamin, Gatenbee Chandler, Robertson-Tessi Mark, Barnes Chris P., Anderson Alexander R. A., Sottoriva Andrea, Graham Trevor A.. Evolutionary dynamics of neoantigens in growing tumours. bioRxiv 536433, doi:10.1101/536433 (2019). PMC7610467 32929288

[39] Levine J. H. Data-Driven Phenotypic Dissection of AML Reveals Progenitor-like Cells that Correlate with Prognosis. Cell 162, 184–197, doi:10.1016/j.cel1.2015.05.047 (2015). 26095251 PMC4508757

[40] Jewer M. Translational control of breast cancer plasticity. Nat Commun 11, 2498, doi:10.1038/s41467-020-16352-z (2020). 32427827 PMC7237473

[41] Quintanal-Villalonga A. Lineage plasticity in cancer: a shared pathway of therapeutic resistance. Nat Rev Clin Oncol 17, 360–371, doi:10.1038/S41571-020-0340-z (2020). 32152485 PMC7397755

[42] Horn L. A., Fousek K. & Palena C. Tumor Plasticity and Resistance to Immunotherapy. Trends Cancer, 6, 432–441, doi:10.1016/j.trecan.2020.02.001 (2020). 32348738 PMC7192950

[43] Arozarena I. & Wellbrock C. Phenotype plasticity as enabler of melanoma progression and therapy resistance. Nat Rev Cancer 19, 377–391, doi:10.1038/S41568-019-0154-4 (2019). 31209265

[44] Doherty M. R., Smigiel J. M., Junk D. J. & Jackson M. W. Cancer Stem Cell Plasticity Drives Therapeutic Resistance. Cancers (Basel) 8, doi:10.3390/cancers8010008 (2016). PMC4728455 26742077

[45] Thiery J. P, Acloque H., Huang R. Y. & Nieto M. A. Epithelial-mesenchymal transitions in development and disease. Cell 139, 871–890, doi:10.1016/j.cel1.2009.11.007 (2009). 19945376

[46] Stemmier M. P., Eccles R. L., Brabletz S. & Brabletz T. Non-redundant functions of EMT transcription factors. Nat Cell Biol 21, 102–112, doi:10.1038/S41556-018-0196-y (2019). 30602760

[47] Balachandran V. P. Identification of unique neoantigen qualities in long-term survivors of pancreatic cancer. Nature 551, 512–516, doi:10.1038/nature24462 (2017). 29132146 PMC6145146

[48] Brown S. D. Neo-antigens predicted by tumor genome meta-analysis correlate with increased patient survival. Genome Res 24, 743–750, doi:10.1101/gr.l65985.113 (2014). 24782321 PMC4009604

[49] Matsushita H. Neoantigen Load, Antigen Presentation Machinery, and Immune Signatures Determine Prognosis in Clear Cell Renal Cell Carcinoma. Cancer Immunol Res 4, 463–471, doi:10.1158/2326-6066.CIR-15-0225 (2016). 26980598

[50] Le D. T. PD-1 Blockade in Tumors with Mismatch-Repair Deficiency. N Engl J Med 372, 2509–2520, doi:10.1056/NEJMoa1500596 (2015). 26028255 PMC4481136

[51] Snyder A. Genetic basis for clinical response to CTLA-4 blockade in melanoma. N Engl J Med 371, 2189–2199, doi:10.1056/NEJMoa1406498 (2014). 25409260 PMC4315319

[52] McGranahan N. Clonal neoantigens elicit T cell immunoreactivity and sensitivity to immune checkpoint blockade. Science 351, 1463–1469, doi:10.1126/science.aaf1490 (2016). 26940869 PMC4984254

[53] Rizvi N. A. Cancer immunology. Mutational landscape determines sensitivity to PD-1 blockade in non-small cell lung cancer. Science 348, 124–128, doi:10.1126/science.aaal348 (2015). 25765070 PMC4993154

[54] Kojima T. Randomized Phase III KEYNOTE-181 Study of Pembrolizumab Versus Chemotherapy in Advanced Esophageal Cancer. J Clin Oncol 38, 4138–4148, doi:10.1200/JCO.20.01888 (2020). 33026938

[55] Sun J. M. Pembrolizumab plus chemotherapy versus chemotherapy alone for first-line treatment of advanced oesophageal cancer (KEYNOTE-590): a randomised, placebo-controlled, phase 3 study. Lancet 398, 759–771, doi: 10.1016/S0140-6736(21)01234-4 (2021). 34454674

[56] Kelly R. J. The Dynamic and Transient Immune Microenvironment in Locally Advanced Esophageal Adenocarcinoma Post Chemoradiation. Ann Surg 268, 992–999, doi:10.1097/SLA.0000000000002410 (2018). 28806299

[57] Kelly R. J. Adjuvant Nivolumab in Resected Esophageal or Gastroesophageal Junction Cancer. N Engl J Med 334, 1191–1203, doi:10.1056/NEJMoa2032125 (2021). 33789008

[58] Shapiro J. Neoadjuvant chemoradiotherapy plus surgery versus surgery alone for oesophageal or junctional cancer (CROSS): long-term results of a randomised controlled trial. Lancet Oncol 16, 1090–1098, doi:10.1016/S1470-2045(15)00040-6 (2015). 26254683

[59] Ergin B. Proteomic analysis of PAXgene-fixed tissues. J Proteome Res 9, 5188–5196, doi:10.1021/pr100664e (2010). 20812734

[60] Groelz D. Non-formalin fixative versus formalin-fixed tissue: a comparison of histology and RNA quality. Exp Mol Pathol 94, 188–194, doi:10.1016/j.yexmp.2012.07.002 (2013). 22814231

[61] Becker K. Histomorphology and grading of regression in gastric carcinoma treated with neoadjuvant chemotherapy. Cancer 98, 1521–1530, doi:10.1002/cncr.11660 (2003). 14508841

[62] Jiang H., Lei R., Ding S. W. & Zhu S. Skewer: a fast and accurate adapter trimmer for next-generation sequencing paired-end reads. BMC Bioinformatics 15, 182, doi:10.1186/1471-2105-15-182 (2014). 24925680 PMC4074385

[63] Schneider V. A. Evaluation of GRCh38 and de novo haploid genome assemblies demonstrates the enduring quality of the reference assembly. Genome Res 27, 849–864, doi:10.1101/gr.213611.116 (2017). 28396521 PMC5411779

[64] Li H. & Durbin R. Fast and accurate short read alignment with Burrows-Wheeler transform. Bioinformatics 25, 1754–1760, doi:10.1093/bioinformatics/btp324 (2009). 19451168 PMC2705234

[65] Van der Auwera G. A. From FastQ data to high confidence variant calls: the Genome Analysis Toolkit best practices pipeline. Curr Protoc Bioinformatics 43, 11 10 11–11 10 33, doi:10.1002/0471250953.bi1110s43 (2013). PMC4243306 25431634

[66] McKenna A. The Genome Analysis Toolkit: a MapReduce framework for analyzing next-generation DNA sequencing data. Genome Res 20, 1297–1303, doi:10.1101/gr.l07524.110 (2010). 20644199 PMC2928508

[67] DePristo M. A. A framework for variation discovery and genotyping using next-generation DNA sequencing data. Nat Genet 43, 491–498, doi:10.1038/ng.806 (2011). 21478889 PMC3083463

[68] Okonechnikov K., Conesa A. & Garcia-Alcalde F. Qualimap 2: advanced multi-sample quality control for high-throughput sequencing data. Bioinformatics 32, 292–294, doi:10.1093/bioinformatics/btv566 (2016). 26428292 PMC4708105

[69] Favero F. Sequenza: allele-specific copy number and mutation profiles from tumor sequencing data. Ann Oncol 26, 64–70, doi:10.1093/annonc/mdu479 (2015). 25319062 PMC4269342

[70] Cibulskis K. Sensitive detection of somatic point mutations in impure and heterogeneous cancer samples. Nat Biotechnol 31, 213–219, doi:10.1038/nbt.2514 (2013). 23396013 PMC3833702

[71] Wang K., Li M. & Hakonarson H. ANNOVAR: functional annotation of genetic variants from high-throughput sequencing data. Nucleic Acids Res 38, e164, doi:10.1093/nar/gkq603 (2010). 20601685 PMC2938201

[72] Khanna A. Bam-readcount – rapid generation of basepair-resolution sequence metrics. ArXiv(2021). 10.21105/joss.03722 PMC13367885 42453900

[73] Martinez-Jimenez F. A compendium of mutational cancer driver genes. Nat Rev Cancer 20, 555–572, doi:10.1038/s41568-020-0290-x (2020). 32778778

[74] Caravagna G., Sanguinetti G., Graham T. A. & Sottoriva A. The MOBSTER R package fortumour subclonal deconvolution from bulk DNA whole-genome sequencing data. BMC Bioinformatics 21, 531, doi:10.1186/si2859-020-03863-1 (2020). 33203356 PMC7672894

[75] Nixon K. C. The Parsimony Ratchet, a New Method for Rapid Parsimony Analysis. Cladistics 15, 407–414, doi:10.1111/j.1096-0031.1999.tb00277.x (1999). 34902938

[76] Schliep K. P. phangorn: phylogenetic analysis in R. Bioinformatics 27, 592–593, doi:10.1093/bioinformatics/btq706 (2011). 21169378 PMC3035803

[77] Swofford D. L. M., Reconstructing W. P. Reconstructing ancestral character states under Wagner parsimony. Math. Biosci 87, 199–229 (1987).

[78] Farris J. S. Methods for Computing Wagner Trees. Syst. Zool. 19, 83 (1970).

[79] Alexandrov L. B. Signatures of mutational processes in human cancer. Nature 500, 415–421, doi:10.1038/nature12477 (2013). 23945592 PMC3776390

[80] Alexandrov L. B., Nik-Zainal S., Wedge D. C., Campbell P. J. & Stratton M. R. Deciphering signatures of mutational processes operative in human cancer. Cell Rep 3, 246–259, doi:10.1016/j.celrep.2012.12.008 (2013). 23318258 PMC3588146

[81] Martincorena I. Universal Patterns of Selection in Cancer and Somatic Tissues. Cell 171, 1029–1041 e1021, doi:10.1016/j.cell.2017.09.042 (2017). 29056346 PMC5720395

[82] Szolek A. OptiType: precision HLA typing from next-generation sequencing data. Bioinformatics 30, 3310–3316, doi:10.1093/bioinformatics/btu548 (2014). 25143287 PMC4441069

[83] Shukla S. A. Comprehensive analysis of cancer-associated somatic mutations in class I HLA genes. Nat Biotechnol 33, 1152–1158, doi:10.1038/nbt.3344 (2015). 26372948 PMC4747795

[84] McGranahan N. Allele-Specific HLA Loss and Immune Escape in Lung Cancer Evolution. Cell 171, 1259–1271 e1211, doi:10.1016/j.cell.2017.10.001 (2017). 29107330 PMC5720478

[85] Alsaab H. O. PD-1 and PD-L1 Checkpoint Signaling Inhibition for Cancer Immunotherapy: Mechanism, Combinations, and Clinical Outcome. Front Pharmacol 8, 561, doi:10.3389/fphar.2017.00561 (2017). 28878676 PMC5572324

[86] Kim S. Strelka2: fast and accurate calling of germline and somatic variants. Nat Methods 15, 591–594, doi:10.1038/s41592-018-0051-x (2018). 30013048

[87] Schenck R. O., Lakatos E., Gatenbee C., Graham T. A. & Anderson A. R. A. NeoPredPipe: high-throughput neoantigen prediction and recognition potential pipeline. BMC Bioinformatics 20, 264, doi:10.1186/s12859-019-2876-4 (2019). 31117948 PMC6532147

[88] s-andrews. FastQC: A quality control analysis tool for high throughput sequencing data, https, <https://github.com/s-andrews/FastQC> (

[89] Dobin A. STAR: ultrafast universal RNA-seq aligner. Bioinformatics 29, 15–21, doi:10.1093/bioinformatics/bts635 (2013). 23104886 PMC3530905

[90] Picard Tools, <http://broadinstitute.github.io/picard/.> (

[91] Love M. I., Huber W. & Anders S. Moderated estimation of fold change and dispersion for RNA-seq data with DESeq2. Genome Biol 15, 550, doi:10.1186/s13059-014-0550-8 (2014). 25516281 PMC4302049

[92] Robinson M. D., McCarthy D. J. & Smyth G. K. edgeR: a Bioconductor package for differential expression analysis of digital gene expression data. Bioinformatics 26, 139–140, doi:10.1093/bioinformatics/btp616 (2010). 19910308 PMC2796818

[93] Hanzelmann S., Castelo R. & Guinney J. GSVA: gene set variation analysis for microarray and RNA-seq data. BMC Bioinformatics 14, 7, doi:10.1186/1471-2105-14-7 (2013). 23323831 PMC3618321

[94] Yu G., Wang L. G., Han Y. & He Q. Y. clusterProfiler: an R package for comparing biological themes among gene clusters. OMICS 16, 284–287, doi:10.1089/omi.2011.0118 (2012). 22455463 PMC3339379

[95] Schwabenland M. Deep spatial profiling of human COVID-19 brains reveals neuroinflammation with distinct microanatomical microglia-T-cell interactions, Immunity 54, 1594–1610 e1511, doi:10.1016/j.immuni.2021.06.002 (2021). 34174183 PMC8188302

[96] Berg S. ilastik: interactive machine learning for (bio)image analysis. Nat Methods 16, 1226–1232, doi: 10.1038/s41592-019-0582-9 (2019). 31570887

[97] Stirling D. R. CellProfiler 4: improvements in speed, utility and usability. BMC Bioinformatics 22, 433, doi:10.1186/s12859-021-04344-9 (2021). 34507520 PMC8431850

[98] Uddin I. Quantitative analysis of the T cell receptor repertoire. Methods Enzymol 629, 465–492, doi:10.1016/bs.mie.2019.05.054 (2019). 31727254

[99] Oakes T. The T Cell Response to the Contact Sensitizer Paraphenylenediamine Is Characterized by a Polyclonal Diverse Repertoire of Antigen-Specific Receptors. Front Immunol 8, 162, doi:10.3389/fimmu.2017.00162 (2017). 28261218 PMC5311069

